# The Brittle Rachis Trait in Species Belonging to the Triticeae and Its Controlling Genes *Btr1* and *Btr2*

**DOI:** 10.3389/fpls.2020.01000

**Published:** 2020-07-22

**Authors:** Xiaoxue Zeng, Kohei Mishina, Juqing Jia, Assaf Distelfeld, Peter Jeff Maughan, Shinji Kikuchi, Hidenori Sassa, Takao Komatsuda

**Affiliations:** ^1^Institute of Crop Science, National Agriculture and Food Research Organization (NARO), Tsukuba, Japan; ^2^Graduate School of Horticulture, Chiba University, Matsudo, Japan; ^3^School of Plant Sciences and Food Security, Tel Aviv University, Tel Aviv, Israel; ^4^Department of Plant & Wildlife Sciences, Brigham Young University, Provo, UT, United States

**Keywords:** seed dispersal, disarticulation, phylogeny, duplication, Triticeae

## Abstract

In many non-cultivated angiosperm species, seed dispersal is facilitated by the shattering of the seed head at maturity; in the Triticeae tribe, to which several of the world's most important cereals belong, shattering takes the form of a disarticulation of the rachis. The products of the genes *Btr1* and *Btr2* are both required for disarticulation to occur above the rachis nodes within the genera *Hordeum* (barley) and *Triticum/Aegilops* (wheat). Here, it has been shown that both *Btr1* and *Btr2* are specific to the Triticeae tribe, although likely paralogs (*Btr1-like* and *Btr2-like*) are carried by the family Poaceae including Triticeae*. Aegilops tauschii* (the donor of the bread wheat D genome) lacks a copy of *Btr1* and disarticulation in this species occurs below, rather than above the rachis node; thus, the product of *Btr1* appears to be required for disarticulation to occur above the rachis node.

## Introduction

Shattering of the seed head at maturity has evolved as an effective means of seed dispersal in many angiosperms and represents one of the most conspicuous differences between a wild species and its related domesticate(s) (Zohary et al., 2012; Olsen and Wendel, 2013; Dong and Wang, 2015). Since shattering does not allow harvesting to be carried out after physiological maturity, the selection of non-shattering types is considered to be a key crop domestication event.

**Figure 1:**
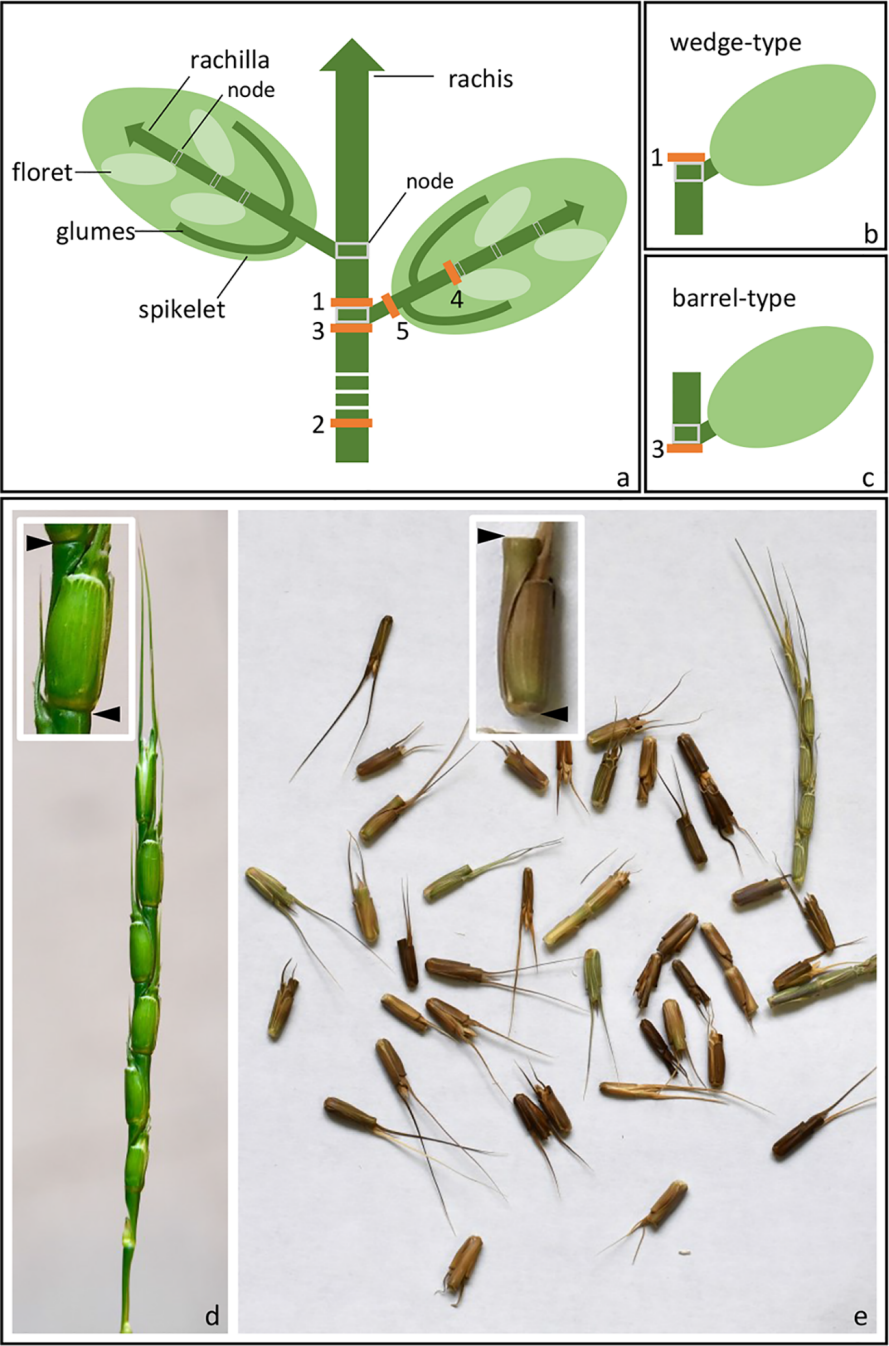
Spike disarticulation in Triticeae species. **(A)** In the mature spike, disarticulation can occur either (1) above the rachis nodes, (2) above the lowest rachis node, (3) below the rachis nodes, (4) below the rachilla nodes, or (5) below the glumes. **(B)** Disarticulation above the rachis node produces a wedge-type dispersal unit, while **(C)** disarticulation below the rachis node produces a barrel-type dispersal unit. **(D, E)** Barrel-type brittle rachis generated from *Ae. tauschii* (accession: AL8/78). Spike before **(D)** and upon **(E)** maturity was shown. Arrows indicate the disarticulation point.

The Triticeae tribe harbors a number of the most important cereal crop species, including barley (*Hordeum vulgare*), cereal rye (*Secale cereale*), and the various forms of wheat, including diploid einkorn (*Triticum monococcum*), tetraploid emmer (*T. turgidum* ssp. *dicoccum*), and durum (*T. turgidum* ssp*. durum*) and hexaploid bread (*T. aestivum*). Seed dispersal in wild Triticeae species is achieved by a process of disarticulation affecting various parts of the mature inflorescence (Frederiksen and Seberg, 1992; Sakuma et al., 2011). In “brittle rachis” types, the flower stalk disarticulates at a number of sites, while in “brittle rachilla” types, it occurs instead along the axis of the spikelet (**Figure 1**, **Table 1**). Both types are represented within each of the 32 genera belonging to the Triticeae tribe (Sakuma et al., 2011). The site of rachis disarticulation varies from species to species (**Figure 1A**): when it occurs above a rachis node, “wedge-type” dispersal units are formed (**Figure 1B**): these feature commonly among the wild relatives of the cereals, such as in *H. vulgare* ssp. *spontaneum* (the ancestor of domesticated barley), in *T. dicoccoides* (the ancestor of cultivated polyploidy wheats), in *S. vavilovii* (the ancestor of cereal rye) and in both *T. monococcum* ssp. *boeoticum* (the ancestor of einkorn wheat) and *T. urartu* (the donor of the A genome present in both durum and bread wheat) (Harlan and Zohary, 1966; Zohary et al., 2012). Breakage at a single site above the lowermost rachis node results in a “whole spike-type” or “umbrella-type” dispersal unit, while its occurrence below a rachis node results in the “barrel-type” dispersal units produced by *Ae. tauschii*, the donor of bread wheat's D genome (**Figure 1C**). Rachilla disarticulation can occur at two sites (**Figure 1A**): the most common phenotype involves breakage below every rachilla node, resulting in dispersal units similar to those produced by *Ae. tauschii*. Disarticulation below the glumes is rare in the Triticeae: the only known example is in *Elytrigia repens* (Sakuma et al., 2011); on the other hand, it is frequent among species belonging to the tribes Oryzeae, Paniceae and Andropogoneae (**Table 1**).

**Table 1:** Seed shattering characterized in the set of wild Poaceae species.

Subfamily	Tribe	Species	2n	Genome	Rachis brittleness	Rachilla brittleness	Disarticulation zone	Dispersal unit (shape)	Reference
Pooideae	Triticeae	*Hordeum vulgare* ssp. *spontaneum*	14	H	Brittle	Non-brittle	Above rachis nodes	Spikelet and rachis segment (wedge)	Zohary et al., 2012
Pooideae	Triticeae	*Secale vavilovii*	14	R	Brittle	Non-brittle	Above rachis nodes	Spikelet and rachis segment (wedge)	Zohary et al., 2012
Pooideae	Triticeae	*Triticum monococcum* ssp. *boeoticum*	14	A	Brittle	Non-brittle	Above rachis nodes	Spikelet and rachis segment (wedge)	Zohary et al., 2012
Pooideae	Triticeae	*Triticum urartu*	14	A	Brittle	Non-brittle	Above rachis nodes	Spikelet and rachis segment (wedge)	Zohary et al., 2012
Pooideae	Triticeae	*Triticum turgidum* ssp. *dicoccoides*	28	AB	Brittle	Non-brittle	Above rachis nodes	Spikelet and rachis segment (wedge)	Zohary et al., 2012
Pooideae	Triticeae	*Aegilops sharonensis*	14	S	Brittle	Non-brittle	Above rachis nodes	Spikelet and rachis segment (wedge)	van Slageren, 1994
Pooideae	Triticeae	*Aegilops longissima*	14	S	Brittle	Non-brittle	Above rachis nodes	Whole spike (umbrella)^1^	van Slageren, 1994
Pooideae	Triticeae	*Aegilops speltoides* ssp. *speltoides*	14	S	Brittle	Non-brittle	Above rachis node	Whole spike (umbrella)^2^	van Slageren, 1994
Pooideae	Triticeae	*Aegilops speltoides* ssp. *ligustica*	14	S	Brittle	Non-brittle	Above rachis nodes	Spikelet and rachis segment (wedge)	van Slageren, 1994
Pooideae	Triticeae	*Aegilops tauschii* ssp. *strangulata*	14	D	Brittle	Non-brittle	Below rachis nodes	Spikelet and rachis segment (barrel)	van Slageren, 1994
Pooideae	Poeae	*Lolium perenne*	14	n.a.	Non-brittle	Brittle	Below rachilla nodes	Floret and rachilla segment	Elgersma et al., 1988
Pooideae	Aveneae	*Avena eriantha*	14	n.a.	Non-brittle	Brittle	Below rachilla nodes	Floret and rachilla segment	Delipavlov, 1999
Pooideae	Brachypodieae	*Brachypodium distachyon*	10	n.a.	Non-brittle	Brittle	Below rachilla nodes	Floret and rachilla segment	Opanowicz et al., 2008
Oryzoideae	Oryzeae	*Oryza sativa* ssp. *indica*	24	n.a.	Non-brittle	Brittle	Below glumes	Spikelet	Konishi et al., 2006
Panicoideae	Paniceae	*Setaria viridis*	18	n.a.	Non-brittle	Brittle	Below glumes	Spikelet	Doust et al., 2014
Panicoideae	Andropogoneae	*Sorghum virgatum*	20	n.a.	Non-brittle	Brittle	Below glumes	Spikelet	Lin et al., 2012
Panicoideae	Andropogoneae	*Zea mays* ssp. *parviglumis*	20	n.a.	Brittle	Non-brittle	Below rachis nodes	Spikelet and rachis segment (barrel)	Studer et al., 2017

n.a., Not applicable.

1 Described as “disarticulating as one unit at maturity with the rudimentary or a few lower, fertile spikelets remining attached to the culm” in van Slageren (1994).

2 Described as “disarticulating at maturity as one unit with the rudimentary spikeletes remaining attached to the culm” in van Slageren (1994).

The formation of wedge-type dispersal units is genetically determined in barley by the genes *Btr1* and *Btr2*, a pair of dominant, complementary, linked genes mapping to the short arm of chromosome 3H. The products of *Btr1* and *Btr2* are, respectively, a 196 and a 202 residue protein, the function of neither of which is currently known. A 1 bp deletion in the *Btr1* coding sequence, and one of 11 bp in the *Btr2* coding sequence are sufficient to convert a shattering to a non-shattering (“non-brittle”) phenotype (Pourkheirandish et al., 2015). In *T. monococcum* ssp. *boeoticum*, the substitution of a single residue in the *Btr1* product converts a brittle to a non-brittle rachis (Pourkheirandish et al., 2018; Zhao et al., 2019). In the polyploid wheat species, mutations at both the A and B genome copies of *Btr1* are required for the formation of a non-brittle rachis (Avni et al., 2017). *Btr* homoeoloci have been mapped to regions syntenous with the 3H region in a number of species (Urbano et al., 1988; King et al., 1997; Chen et al., 1998; Li and Gill, 2006; Jiang et al., 2014). Of the 22 species belonging to the genus *Aegilops* (van Slageren, 1994), *Ae. speltoides* is of particular interest in connection with spike disarticulation, because it features two morphological forms of rachis brittleness: while ssp. *speltoides* forms whole spike-type disarticulation units, ssp. *ligustica* disarticulates at each rachis node to produce wedge-type ones (Li and Gill, 2006). The whole spike-type disarticulation trait is recessive to the wedge-type one, assumed to reflect an allelic interaction at *Brt1* (Li and Gill, 2006). *Ae. tauschii* exceptionally produces barrel-type dispersal units (van Slageren, 1994). Rather than mapping to the short arm of 3D, however, the locus responsible for this trait maps to the long arm of the chromosome as shown in three independent crosses (Amagai et al., 2015; Katkout et al., 2015; Zhang et al., 2015).

The evolutionary history of *Btr1* and *Btr2* remains obscure. The barley genome harbors a paralog of both *Btr1* and *Btr2* (respectively, *Btr1-like* and *Btr2-like*); while all four of these genes map to 3HS, *Btr1-like* and *Btr2-like* are separated from one another by just 4.2 kbp, but *Btr2* maps 100 kbp away from this locus and *Btr1* 200 kbp away (Pourkheirandish et al., 2015). A similar situation pertains in *T. turgidum* (Avni et al., 2017). The relevant duplication events are known to have occurred post the divergence of the *Hordeum* and *Brachypodium* lineages (Pourkheirandish et al., 2015). The aim of the present study was to shed more light on the evolutionary events surrounding the acquisition in the Triticeae of *Btr1* and *Btr2*.

## Materials and Methods

### Plant Materials

A stock of *T. monococcum* ssp. *boeoticum* (accession KT1-1) was obtained from the National BioResource Project (NBRP)/KOMUGI, Kyoto University, Kyoto, Japan and used for PCR-cloning. Grains of *S. vavilovii* were kindly provided by Prof. Eva Bauer, Technische Universität München, Munich, Germany and used for phenotype observation and PCR-cloning. All the materials were grown in a greenhouse at NARO (Tsukuba, Japan).

### Identification of Sequences Homologous to *Btr*

The coding sequences of *Btr1* (591 nt), *Btr1-like* (597 nt), *Btr2* (609 nt) and *Btr2-like* (579 nt), all housed on the *H. vulgare* ssp. *spontaneum* (accession OUH602) BAC clone KR813335.1 (Pourkheirandish et al., 2015), were used as query sequences for a BLASTn search (E-value threshold: 1E−20) of the genomic sequences of *S. vavilovii* (Bauer et al., 2017), *T. urartu* (Ling et al., 2018), *Ae. sharonensis*, *T. aestivum* (Alaux et al., 2018), and *Ae. speltoides* ssp. *speltoides* (unpublished data provided by A Distelfeld [Tel Aviv University, Israel]), *Ae. longissima* (unpublished data provided by A Sharon [Tel Aviv University]), *Ae. tauschii* (Luo et al., 2017), *T. dicoccoides* (Avni et al., 2017), *Lolium perenne* (Byrne et al., 2015), *Avena eriantha* (provided by Maughan et al., 2019, [Brigham Young University, Provo, UT, USA]), and *Brachypodium distachyon* (International Brachypodium Initiative, 2010). A BLASTp search (E-value threshold: 1E-2 and identity threshold: 30%) was used to identify homologs present in rice (Kawahara et al., 2013), foxtail millet (*Setaria italica*) (Bennetzen et al., 2012), sorghum (Paterson et al., 2009), maize (Jiao et al., 2017), and *Arabidopsis thaliana* (Swarbreck et al., 2008).

### Acquiring the Sequence of *S. vavilovii* and *T. monococcum Btr* Homologs

Genomic DNA was extracted from fresh leaves of *S. vavilovii* and *T. monococcum*, as described by Komatsuda et al. (1998), to provide the template for PCRs driven by primers designed using Primer 3 software (bioinfo.ut.ee/primer3) from their respective genomic DNA contigs (**Table S1**). Each 10 µl PCR contained 0.25 U ExTaq polymerase (Takara, Tokyo, Japan), 1× ExTaq polymerase buffer, 0.3 μM of each primer, 200 μM dNTP, 2 mM MgCl_2_, 2.5% v/v DMSO, and 20 ng genomic DNA. The amplification regime comprised an initial denaturation step (94°C/5 min), followed by 30 cycles of 94°C/30 s, 57°C–62°C (primer pair dependent, see **Table S1**)/30 s, 72°C/90 s, and a final extension step of 72°C/10 min. The resulting amplicons were purified using a QIAquick PCR purification kit (Qiagen, Germantown, MD, USA), and sequenced using a reaction based on Big Dye Terminator v3.1 (Applied Biosystems, Foster City, CA, USA). The Agencourt CleanSeq system (Beckman Coulter Inc., Brea, CA, USA) was used to remove salts, non-incorporated dNTPs and dye terminator, and the sequence data acquired using an ABI Prism 3130/3730xL sequencer (Applied Biosystems).

### Gene and Protein Structure Prediction

The coding region of the *S. vavilovii* and *T. monococcum Btr* homologs was identified using the FGENESH program (linux1.softberry.com/berry.phtml) in conjunction with codon usage in a selection of monocotyledonous species (Solovyev et al., 2006). Multiple alignments of nucleotide and peptide sequences were obtained using the CLC Sequences Viewer v7.8.1 (www.qiagenbioinformatics.com). Predictions of polypeptide secondary structure were made using the SOSUI program (Hirokawa et al., 1998).

### Phylogenetic Analysis

Nucleotide sequences were aligned using the appropriate algorithm provided by the MUSCLE program (Edgar, 2004), as implemented in the Mega v6 software package (Tamura et al., 2013) and the alignments used to conduct a phylogenetic analysis based on the neighbor-joining algorithm (Saitou and Nei, 1987) supported by 1,000 bootstrap replicates (Felsenstein, 1985). Polypeptide sequences were also aligned using the relevant MUSCLE algorithm and once the optimal model had been selected (Nei, 2000), the alignments were used to generate a phylogeny based on the maximum likelihood method; again, the analysis was supported by 1,000 bootstrap replicates.

### Transcriptional Profiling

Archival RNA-seq data capturing the transcriptome of the root, sheath, leaf, spike, stamen, pistil, stem and caryopsis of *Ae. tauschii* ssp. *strangulata* (Jia et al., 2013), were filtered to remove low quality reads using the FASTP program (Chen et al., 2018). Bowtie2 software (Langmead and Salzberg, 2012) was used to align and count the reads. Transcript abundances were expressed in the form reads per kilobases per million reads (RKPM) (Mortazavi et al., 2008), and were averaged where replicated samples were available.

## Results

### *Btr1* Sequences Present in the Grasses

The relevant statistical parameters associated with the genome assemblies are given in **Table S2**. A BLASTn search successfully retrieved *Btr* homologs from a number of members of the Triticeae, Poeae, Aveneae and Brachypodieae tribes (**Supplemental Fasta File**: Btr1_Btr1-like_DNA.fasta and Btr2_Btr2-like_DNA.fasta), as well as from a number of non-Pooideae species (identified using a BLASTp search), but not from *A. thaliana*. Species not belonging to the Triticeae tribe lacked a copy of *Btr1*, but harbored one or more *Btr1-like* genes (one in each of rice, foxtail millet and sorghum, two in *L. perenne*, three in *Av. eriantha* and four in *B. distachyon*). No *Btr1-like* sequence was recovered from maize (**Table S3**). Most of the Triticeae wild species harbored *Btr1-like* genes, with a copy number varying from one to three; none of these genes was interrupted by an intron (**Figure S1**, **Table S3**). Two *Btr1-like* copies were recovered from *T. monococcum* ssp. *boeoticum*: these were *Btr1-like-A-1*, predicted to encode a 195 residue polypeptide and *Btr1-like*-*A-2* (a 194 residue polypeptide) (**Table S3**). The two sequences were 96% identical at the nucleotide level, including 22 polymorphic sites (**Figure S2A**), while their predicted polypeptide sequences shared 91% identity, differing at 17 sites (**Figure S2B**). Given that the species' inbreeding habit ensures a high level of homozygosity, the possibility that these two genes represent alleles is unlikely. The set of *Btr1* sequences clustered within a single phylogenetic clade (the “*Btr1* clade”), which was supported by a bootstrap probability of 99% (**Figure S1**). The remaining related sequences, referred to as ‘*Btr1-like*', also fell into this clade, implying that *Btr1* and *Btr1-like* genes evolved from a single Triticeae sequence, which was later duplicated. The *Btr1* open reading frame was in most cases a 591 bp sequence uninterrupted by introns (**Table S3**), as previously described for the copies present in barley (Pourkheirandish et al., 2015), *T. monococcum* (Pourkheirandish et al., 2018) and *T. turgidum* (Avni et al., 2017).

### Deviations in the Canonical Structure of the *Btr1* Sequence

*S. vavilovii* contig 160742 harbored a sequence homologous to *Btr1* (**Table S3**). Its coding sequence differed from the barley copy with respect to a small deletion involving nucleotides 532 through 535 (**Figure S3A**). In addition, the coding sequence was split into two exons. Its predicted product was somewhat shorter (186 residues) and included a frame shift in its N terminal region (**Figure S3B**). This *S. vavilovii* accession used to generate the whole genome sequence (Bauer et al., 2017), exhibited a brittle rachis (**Figure S4**). To confirm the presence of the 4 nt deletion, its *Btr1* content was PCR-amplified from a template of the relevant *S. vavilovii* accession and the resulting amplicon sequenced. Two distinct *Btr1* copies were recovered: one included the 4 nt deletion, while the other was a complete 591 nt sequence, predicted to encode the same 196 residues as the barley *Btr1* gene does (**Table S3**). The two copies are referred to here as, respectively, *Btr1-R-2* and *-R-1*. The two sequences differed at 18 sites in the coding region (**Figure S3A**); it remains unclear whether *-R-2* and *-R-1* are allelic, or whether they reside at independent loci, but the former is more likely, given that *S. vavilovii* is an out-pollinator.

The *Ae. sharonensis* genome harbored three copies of *Btr1*: one of these (TSL_WGS_sharonensis_v1_contig_98068 [3458-2918]) was a complete 591 nt sequence, predicted to encode 196 residues; the second (TSL_WGS_sharonensis_v1_contig_341236 [279-739]) was interrupted by one intron and was predicted by the FGENESH program to encode a 130 residue protein, lacking the codons lying between nucleotides 172 and 307 (**Figure S5A, B**); the third (TSL_WGS_sharonensis_v1_contig_1151931 [2022-1843]) was also a truncated sequence (**Figure S6A**), predicted to encode a 59 residue protein (lacking the codons beyond position 167) (**Figure S6B**).

The *Ae. longissima* genome harbored four copies of *Btr1:* three of these mapped to sites on chromosome 3S (nucleotides 86738095-86738685, 85162031-85162621 and 85013005-85012418) and are predicted to encode a polypeptide of length, respectively, 196, 196, and 195 residues; the fourth, which was contained within a scaffold not assignable to a specific chromosome, was a truncated sequence similar to the one present on the *Ae. sharonensis* TSL_WGS_sharonensis_v1_contig_1151931 (**Figure S6A, B**).

The *Ae. tauschii* genome lacked an intact copy of *Btr1*. The length of the homologous sequence was only 166 nt, mapping to a site on chromosome 3D (59424083-59424248) close to the position occupied by *Btr1* copies in the other Triticeae species. The sequence aligned with the first 167 nucleotides of barley *Btr1*, and was identical with the truncated copies present in both *Ae. longissima* and *Ae. sharonensis*, except for the absence of the thymine base in the start codon (**Figure S6A**). The sequence was genuine, since a search of three independent genome sequence databases reporting the sequence of the AL8/78 accession (Marcussen et al., 2014; Xie et al., 2017; Zimin et al., 2017) retrieved the same sequence in each case (**Table S4**). An identical sequence was also represented in the assembly of bread wheat chromosome 3D (**Table S4** and **Table S5**).

### The Phylogeny of the Proteins Encoded by *Btr1* and *Btr1-Like* Genes

**Figure 2:**
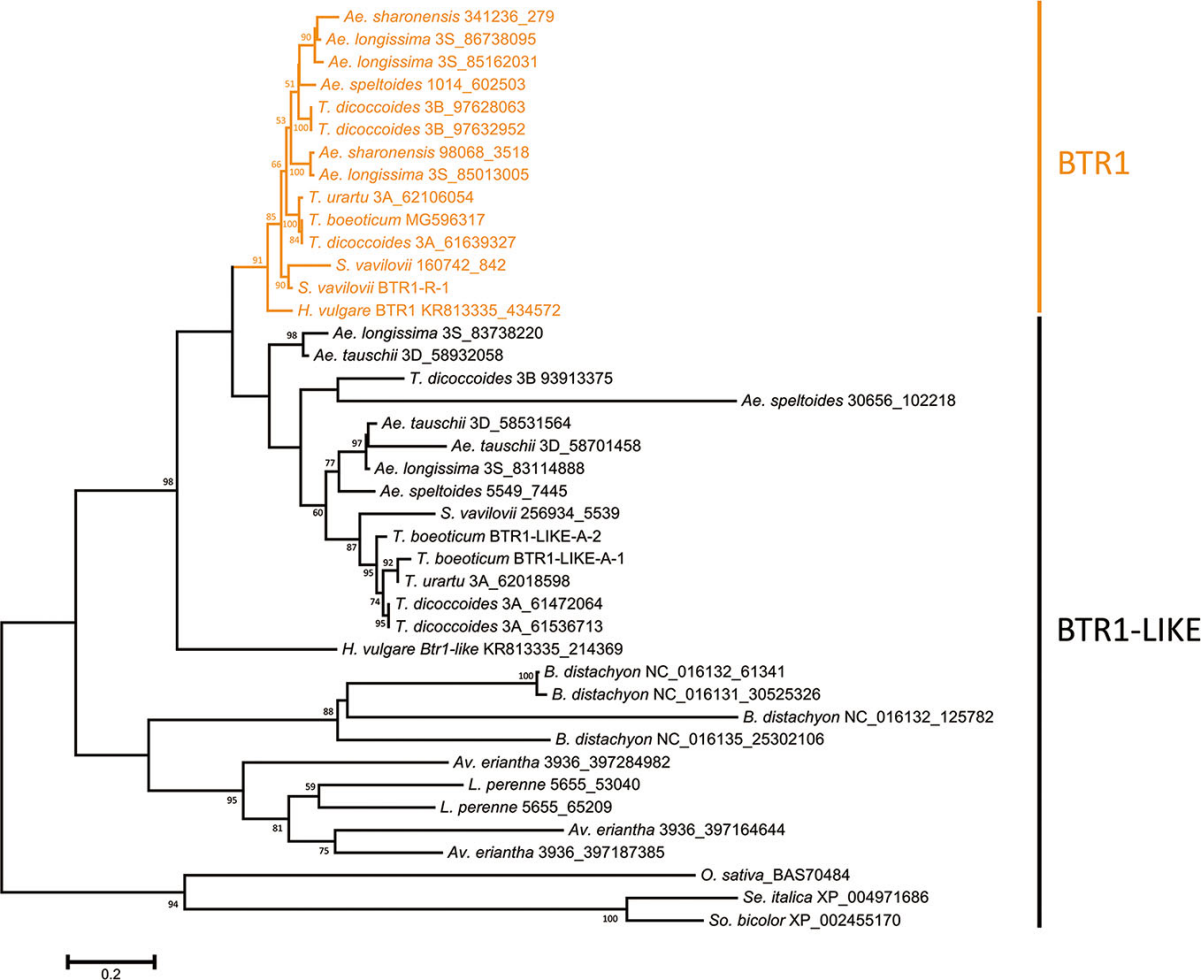
Phylogeny of the Poaceae BTR1 and BTR1-like proteins. The tree was constructed using the maximum likelihood method based on an alignment of deduced polypeptide sequences. The products of the *Btr1* genes are shown in orange. Numbers given at a branch node indicate bootstrap probabilities (only those > 50% are shown). Accession/scaffold/contig numbers and the start location of each gene is given in **Table S3**.

The predicted lengths of the products of the *Btr1* and *Btr1-like* genes harbored by Poaceae species ranged from 170 to 212 residues, while among the Triticeae species, the range was 170–199 (most lay in the range 192–198) (**Table S3**). The majority of *Btr1* genes encoded a 196 residue protein. Consistent with the nucleotide sequence-based phylogeny, two protein clades were recognized in the Triticeae (**Figure 2**). Most of the Triticeae species were found to encode one or more sequences in the BTR1 clade plus one or more in the BTR1-LIKE clade (**Figure 2**). Exceptionally, the *Ae. tauschii* genome encodes no BTR1 proteins, rather harboring three *Btr1-like* genes.

### *Btr2* and *Btr2-Like* Sequences and Their Products

**Figure 3:**
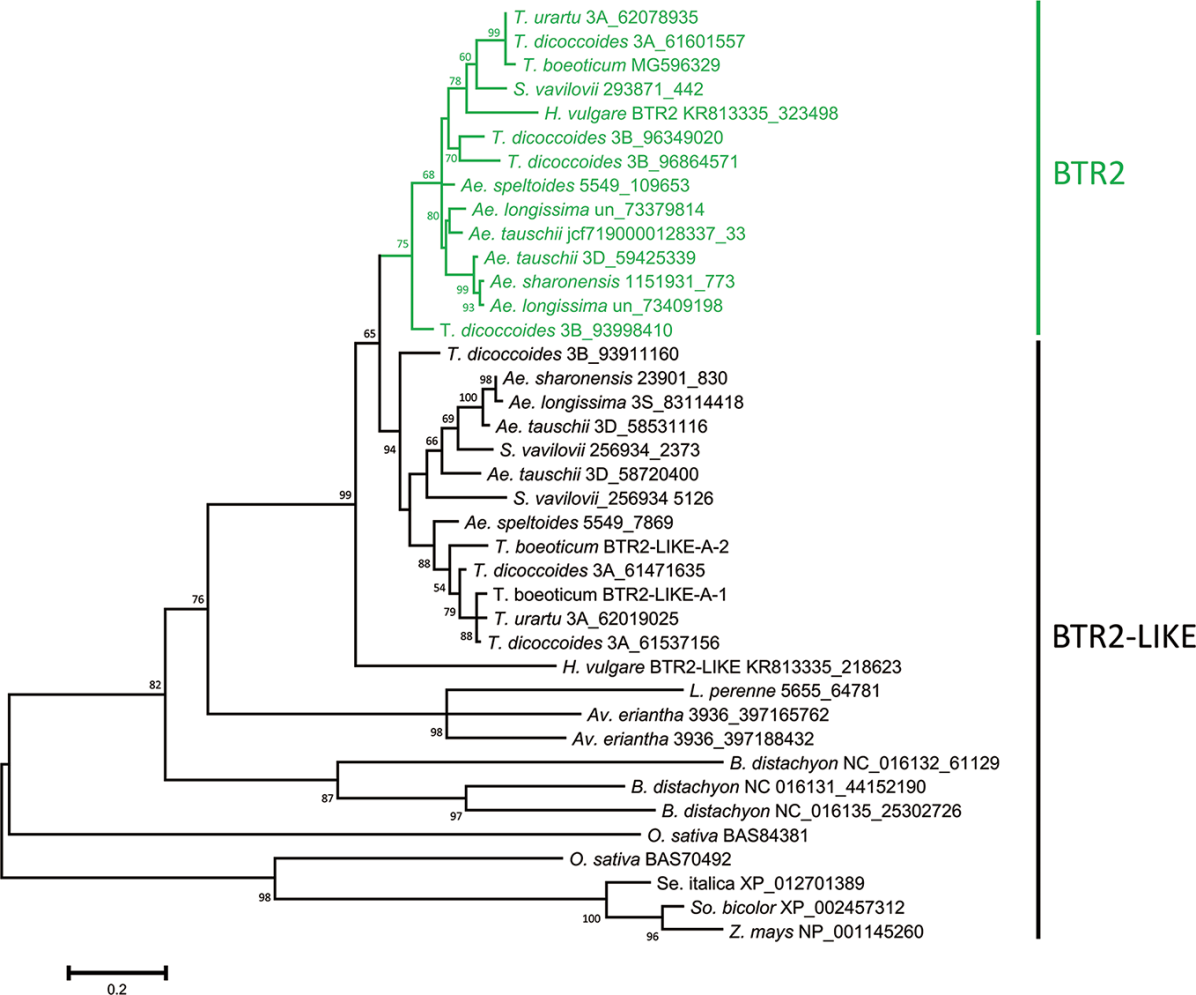
Phylogeny of the Poaceae BTR2 and BTR2-like proteins. The tree was constructed using the maximum likelihood method based on an alignment of deduced polypeptide sequences. The products of the *Btr2* genes are shown in green. Numbers given at a branch node indicate bootstrap probabilities (only those > 50% are shown). Accession/scaffold/contig numbers and the start location of each gene is given in **Table S6**.

The nucleotide sequences classified as either *Btr2* or *Btr2-like* also formed two clades: the “*Btr2* clade” was centered on the barley *Btr2* gene, and was supported by a bootstrap probability of 97% (**Figure S7**). Grass species harbored a variable number of *Btr2-like* sequences. According to a BLASTn search, there was one copy in *L. perenne*, two in *Av. eriantha* and three in *B. distachyon*; while based on a BLASTp search, two copies were located in rice, and one each in foxtail millet, sorghum and maize (**Table S6**). Among the wild Triticeae species, most of the *Btr2* and *Btr2-like* sequences were free of introns, as is also the case for barley *Btr2*. The exceptional case was the *Ae. tauschii* sequence mapping from nucleotides 58720400-58720945 on chromosome 3D, which was split into two exons as predicted by the FGENESH program. *S. vavilovii, T. urartu*, *T. turdigum* ssp. *dicoccoides*, *Ae. sharonensis, Ae. longissima, Ae. speltoides* ssp. *speltoides* and *Ae. tauschii* each harbored at least three *Btr2* or *Btr2-like* sequences per diploid genome (the tetraploid species *T. turgidum* harbored six copies). The predicted length of the set of BTR2 and BTR2-LIKE proteins ranged from 129–426 residues, while among the Triticeae species, the range was much narrower (192–204) (**Table S6**). Two distinct clades were recognized among the Triticeae proteins (**Figure 3**): the one including barley BTR2 (the BTR2 clade) was supported by a bootstrap probability of 75%.

### The Structure of the BTR and BTR-Like Proteins

**Figure 4:**
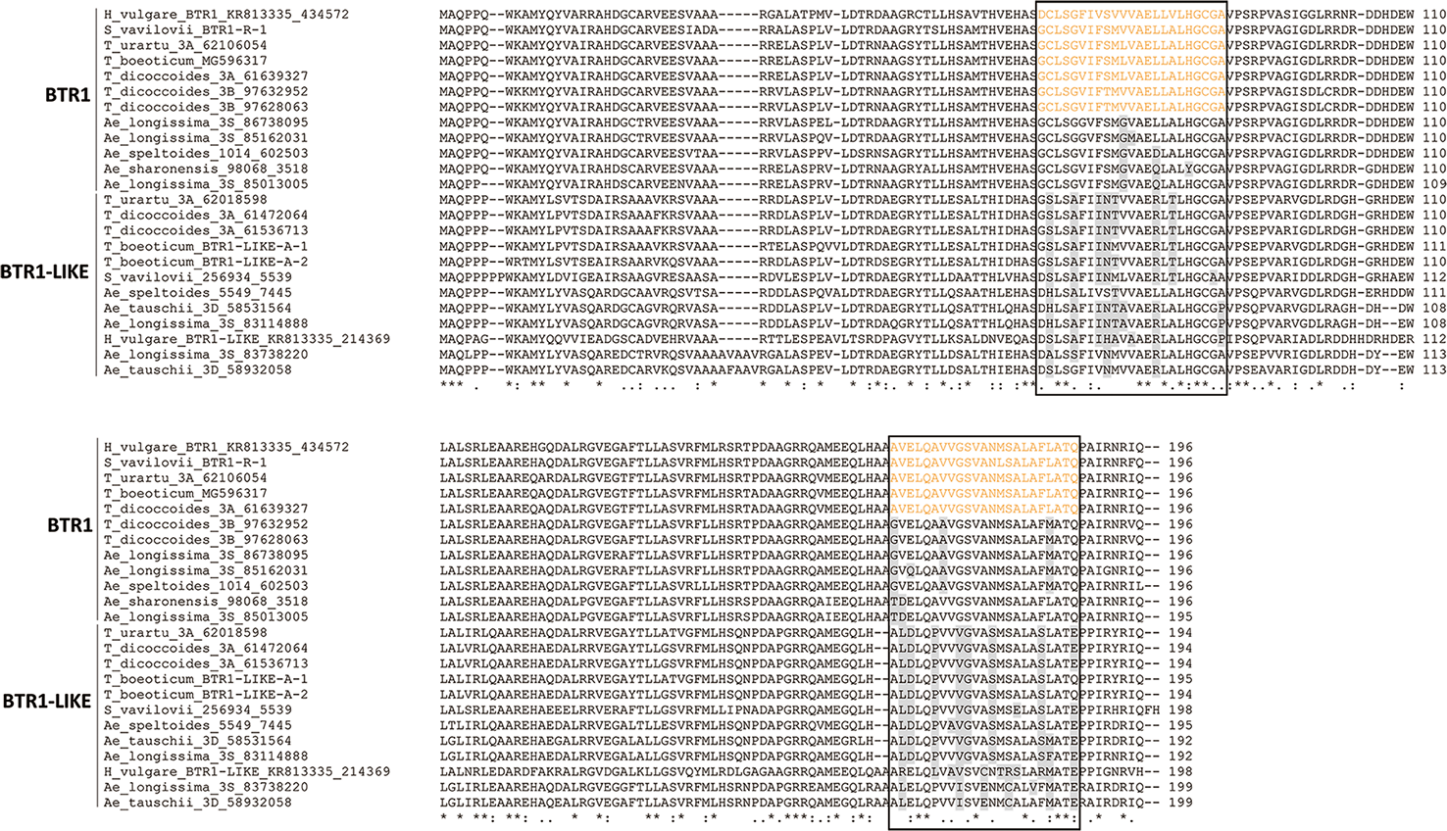
Alignment of deduced Triticeae BTR1 and BTR1-like polypeptides. The two predicted transmembrane helix regions are shown boxed, and the transmembrane helices are colored orange. Variable residues in the transmembrane helices are colored gray.

**Figure 5:**
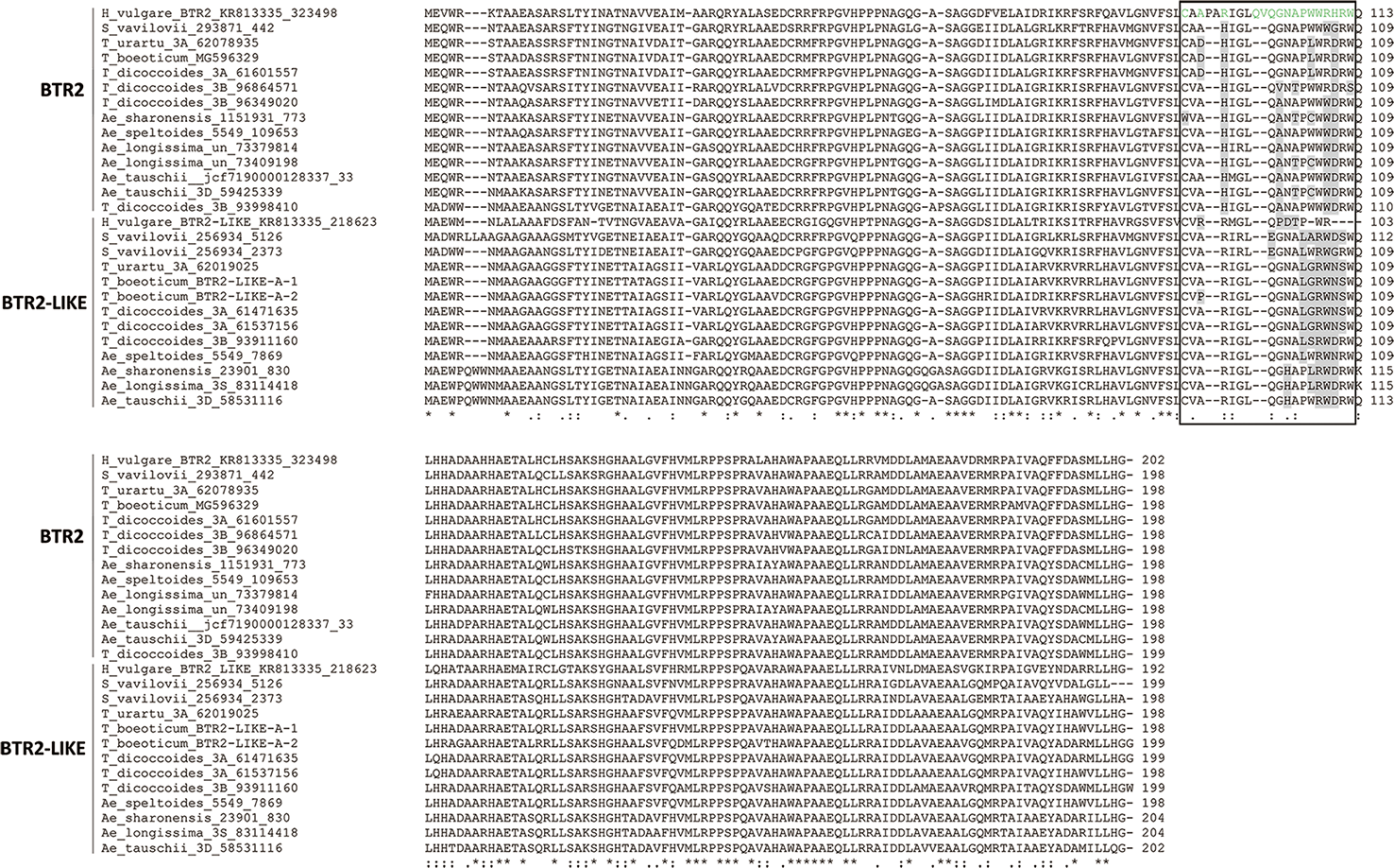
Alignment of deduced Triticeae BTR2 and BTR2-like polypeptides. The CAR-PIP motif region is shown boxed and the motif present uniquely in barley is colored green. Variable residues in the motif region are colored gray.

An alignment of BTR1 and BTR1-LIKE proteins encoded by the Triticeae species is shown in **Figure 4**. One of the two *Ae. speltoides Btr1-like* sequences, namely scaffold_30656 [102218-101631], was excluded because its sequence has clearly experienced a number of deletions. The *Btr1-*encoded proteins produced by *T. urartu*, *T. monococcum* ssp. *boeoticum*, *T. turdigum* ssp. *dicoccoides* (the copy on chromosome 3A), and *S. vavilovii* each featured two transmembrane helices (from residues 66–88 and 166–188), as is also the case for barley BTR1 (Pourkheirandish et al., 2015). The two proteins produced by *T. turdigum* ssp. *dicoccoides* (the copy on chromosome 3B) lacked a second transmembrane helix as a result of polymorphisms at three key residue sites (“G”, “A”, and “M” marked in **Figure 4**). The *Btr1-*encoded proteins produced by *Ae. speltoides, Ae. sharonensis* and *Ae. longissima* lacked any transmembrane helices due to polymorphisms in both domains: one or two in the 66–88 region and two or three in the 166–188 region. The latter polymorphisms in *Ae. speltoides and Ae. longissima* were identical to those present on the chromosome 3B-encoded *T. turdigum* ssp. *dicoccoides* BTR1. Like the barley BTR2 protein (Pourkheirandish et al., 2015), those proteins lacking transmembrane helices were predicted to be soluble, as were all of the Triticeae BTR2 proteins. An alignment of the relevant Triticeae sequences is shown in **Figure 5**. None of these proteins include the CAR-PIP motif present in barley BTR2 (Pourkheirandish et al., 2015), as a result of sequence polymorphisms (**Figure 5**).

### Genomic Organization

**Figure 6:**
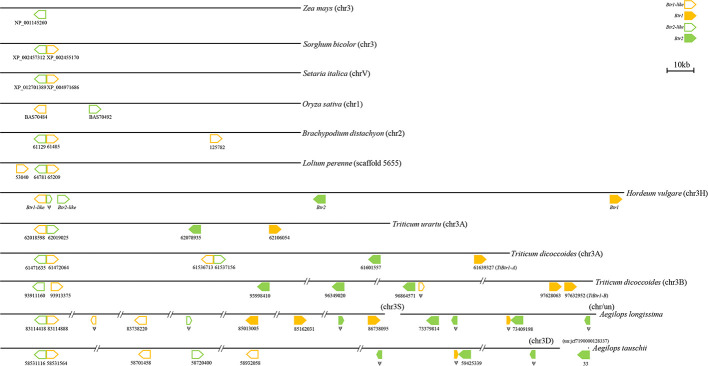
Organization of *Btr1*, *Btr1-like*, *Btr2*, and *Btr2-like* genes in the grasses. The direction of translation is arrowed. Numbers indicate either the gene's ID or the nucleotide positions of the gene's translation start point. Only the chromosome 2 genes of *B. distachyon* are shown.

In species harboring both *Btr1-like* and *Btr2-like* genes, in most cases, the loci lie close to one another: in foxtail millet, the separation is as little as 314 nt. The major exception is in rice, where the genes are separated by 18,000 nt (**Table 2**, **Figure 6**). In each case, the pair of genes is oriented head-to-head. The separation between the *Btr1* and *Btr2* loci is much greater: 111,000 nt in barley, 27,000 nt in *T. urartu*, 37,000 nt in *T. dicoccoides* (3A copy), and 763,000 nt in *T. dicoccoides* (3B copy); again, though, their orientation is consistently head-to-head. The separation between the *Btr1-like* and *Btr2-like* sequences is consistently smaller than between *Btr1* and *Btr2*, because even where the relative location of the latter remains uncertain, at least the two genes each mapped onto a different contig or scaffold (**Tables S3** and **S6**).

**Table 2:** Separation between Poaceae *Btr1-like* and *Btr2-like* genes.

Species	Chromosome/contig/accession	Distance (bp)
*Hordeum vulgare* ssp. *spontaneum*	3H/KR813335.1	4254
*Secale vavilovii*	Svavi_v1_contig_256934	413
*Trticum monococcum* ssp. *boeoticum*	MT586112	435
	MT586113	390
*Triticum urartu*	3A/CM009795.1	427
*Triticum turgidum* ssp*. dicoccoides*	3A	443
*Triticum turgidum* ssp*. dicoccoides*	3A	441
*Triticum turgidum* ssp*. dicoccoides*	3B	2215
*Aegilops longissima*	3S	470
*Aegilops speltoides* ssp. *speltoides*	Scaffold_5549	424
*Aegilops tauschii* ssp. *strangulata*	3D	448
*Lolium perenne*	Scaffold_5655	428
*Brachypodium distachyon*	chr2/NC_016132.3	356
*Oryza sativa*	chr1/AP014957.1	18642
*Setaria italica*	chrV/NC_028454.1	448
*Sorghum bicolor*	chr3/NC_012872.2	332

### Transcriptional Analysis in *Ae. tauschii*

The RPKM value was used to calculate the abundance of *Btr* transcript in *Ae. tauschii*. As shown in **Figure S8**, the two *Btr2* genes are transcribed specifically in young spikes. Meanwhile, the linked pair *Btr1-like* (3D [58531564-58532142]) and *Btr2-like* (3D [58531116-58530508]) are less abundantly transcribed in young spike than the other copy of *Btr1-like* (3D [58932058-58931459]), whereas both were more abundantly transcribed in the stamen. Transcript of each of the orthologs of rice seed shattering genes (with the exception of the *sh4* ortholog) is more abundant than *Btr2*, not only in young spikes but also elsewhere in the plant (**Table S5**, **Figure S9**).

## Discussion

### *Btr1-Like* and *Btr2-Like* Genes Are Conserved in the Poaceae

The present analysis of a sample of Poaceae species showed that copies of both *Btr1-like* and *Btr2-like* have been retained in their expected genomic region, i.e., in the region sharing synteny with barley chromosome 3H (Salse, 2016). This level of conservation implies that their products likely perform an indispensable function. Given that the loci housing the *Btr1-like* and *Btr2-like* sequences lie close to one another (except in rice), and that the orientation of the two genes is invariably head-to-head. One hypothesis is that they are co-regulated, as is the case in *Ae. tauschii* (**Figure S9**), but further experiments are needed to verify it.

### *Btr1* and *Btr2* Evolved in the Tribe Triticeae

It has been demonstrated previously that *Btr1* and *Btr1*-*like* evolved as a result of a duplication event (Pourkheirandish et al., 2015). The *Btr1*-*like* sequence is well conserved in the Poaceae, while *Btr1* is only found in species belonging to the Triticeae tribe. The relationship between *Btr2* and *Btr2*-*like* is similar. Species within the genera *Lolium* and *Avena* lack both *Btr1* and *Btr2*, even though the Poeae and Aveneae tribes are considered to be closely related to the Triticeae (Kellogg, 1998). The suggestion is therefore that the duplication event(s) which led to the appearance of *Btr1* and *Btr2* occurred after the divergence of the Triticeae tribe from the Poeae and the Aveneae tribes. Whether all members of the Triticeae – which harbors some 30 genera (Barkworth and von Bothmer, 2009) - retain both *Btr1* and *Btr2* has yet to be determined.

### BTR1 and BTR2 Are Probably Involved in the Rachis Disarticulation Trait

*Btr1* orthologs are required for disarticulation above the rachis nodes, since the loss-of-function *btr1* mutant forms a non-brittle rachis in both *Hordeum* and *Triticum* spp. (Pourkheirandish et al., 2015; Avni et al., 2017; Pourkheirandish et al., 2018). The present analysis has established that all of the wild Triticeae species which exhibit disarticulation above the rachis nodes carry a copy of *Btr1*. *Ae. speltoides* ssp. *speltoides* is unique in disarticulating only above the lowest rachis node; this species harbors a copy of *Btr1*, so one hypothesis, plausibly testable using *in situ* RNA hybridization and/or transcriptomic profiling of micro-dissected rachis nodes, is that the gene is regulated differently in *Ae. speltoides* ssp. *speltoides* than in species which disarticulate above each rachis node. *Ae. tauschii* lacks an intact copy of *Btr1* and disarticulates below the rachis nodes; the inference is that BTR1 is not required to effect disarticulation below the rachis nodes.

A *Btr2* gene was harbored by each of the Triticeae species examined, implying that its product is involved in the determination of the brittle rachis trait; in particular, the gene was present in species which disarticulate above the rachis nodes. In barley, the finding that *Btr2* expression occurs in a thin cell layer above the rachis node has been taken to imply that BTR2 contributes to the formation of the disarticulation zone (Pourkheirandish et al., 2015). Whether BTR2 in *Ae. tauschii* is involved in the same way below the rachis node remains an open question. However, it is clear that *Btr2* transcript is generated in immature *Ae. tauschii* spikes, although at a rather low abundance (**Figure S8**). Note that *Ae. tauschii* harbors two copies of *Btr2*, so it is possible that one of these is expressed above the rachis nodes, but is inactive since *Ae. tauschii* lacks an intact copy of *Btr1* to induce disarticulation there; meanwhile the second copy is perhaps expressed below the rachis nodes.

### *Ae. tauschii* Lacks a Copy of *Btr1*

*Ae. tauschii* does not harbor an intact copy of *Btr1*, but this gene is not essential for this species, because its rachis disarticulates below the node. It is arguable that *Ae. tauschii* could be an evolutionary intermediate between the Poeae/Aveneae and the Triticeae tribes, since members of the former two tribes also lack *Btr1*. However, unlike members of the Poeae/Aveneae, *Ae. tauschii* does harbor an intact copy of *Btr2*. The argument would require that *Btr2*, and later *Btr1*, were acquired independently, which appears to be less plausible than the suggestion that the *Btr1-like* and *Btr2-like* pair was duplicated, allowing for a later divergence from *Btr1-like* to *Btr1* and *Btr2-like* to *Btr2*, as suggested by Pourkheirandish et al. (2015). An alternative evolutionary pathway can be based on the assumption that the truncated *Btr1* sequences present in *Ae. tauschii* (166 bp), *Ae. sharonensis* (167 bp) and *Ae. longissima* (167 bp) share a common origin. *Ae. tauschii* and *Ae. sharonensis* diverged some 2 Mya (Marcussen et al., 2014), after which *Ae. tauschii* lost its intact copy of *Btr1*, but retained the truncated one; meanwhile both *Ae. sharonensis* and *Ae. longissima* retained both the intact and the truncated *Btr1* sequences. Disarticulation below the rachis nodes could have evolved in *Ae. tauschii* following the *de novo* recruitment (or perhaps neofunctionalization) of a co-operating gene(s). The latter may include orthologs of genes known to be responsible for shattering in rice, such as *qSH1*, *sh4*, *SH5*, *SHAT1*, *CPL1* and *OSH15* (Konishi et al., 2006; Li et al., 2006; Ji et al., 2010; Zhou et al., 2012; Yoon et al., 2014; Yoon et al., 2017), since orthologs of these genes are present in *Ae. tauschii* (**Table S5**), and are transcribed in the immature spike (**Figure S9**). Especially, the *sh4* and *OsCPL1* orthologs showed higher expression than the other ones in the immature spikes of *Ae. tauschii*.

## Data Availability Statement

All datasets presented in this study are included in the article/**Supplementary Material**.

## Author Contributions

XZ and TK planned and designed the research. TK, HS, SK, and JJ supervised the experiments. XZ performed the experiments. KM participated in the phylogenetic analysis and genome informatics; AD and PM analyzed the data and provided advices. XZ and TK wrote the manuscript. All authors contributed to the article and approved the submitted version.

## Funding

This research was supported in part by a grant-in-aid from the Japan Society for the Promotion of Science (JSPS) to TK (18H02184). XZ appreciates the Monbukagakusho scholarship from Japanese Government (MEXT).

## Conflict of Interest

The authors declare that the research was conducted in the absence of any commercial or financial relationships that could be construed as a potential conflict of interest.

The reviewer SS declared a past collaboration with one of the authors TK to the handling Editor.

## Supplementary Material

Data Sheet 1 Btr1_Btr1-like_DNA.fasta.

Data Sheet 2 Btr1_Btr1-like_Protein.fasta.

Data Sheet 3 Btr2_Btr2-like_DNA.fasta.

Data Sheet 4 Btr2_Btr2-like_Protein.fasta.
