# Supplementary material for: The Brittle Rachis Trait in Species Belonging to the Triticeae and Its Controlling Genes Btr1 and Btr2
Source: Front Plant Sci. 2020 Jul 22;11:1000. doi: 10.3389/fpls.2020.01000 (PMC7387508; doi:10.3389/fpls.2020.01000)
Supplement: Supplementary file 5 [file DataSheet_5.pdf]

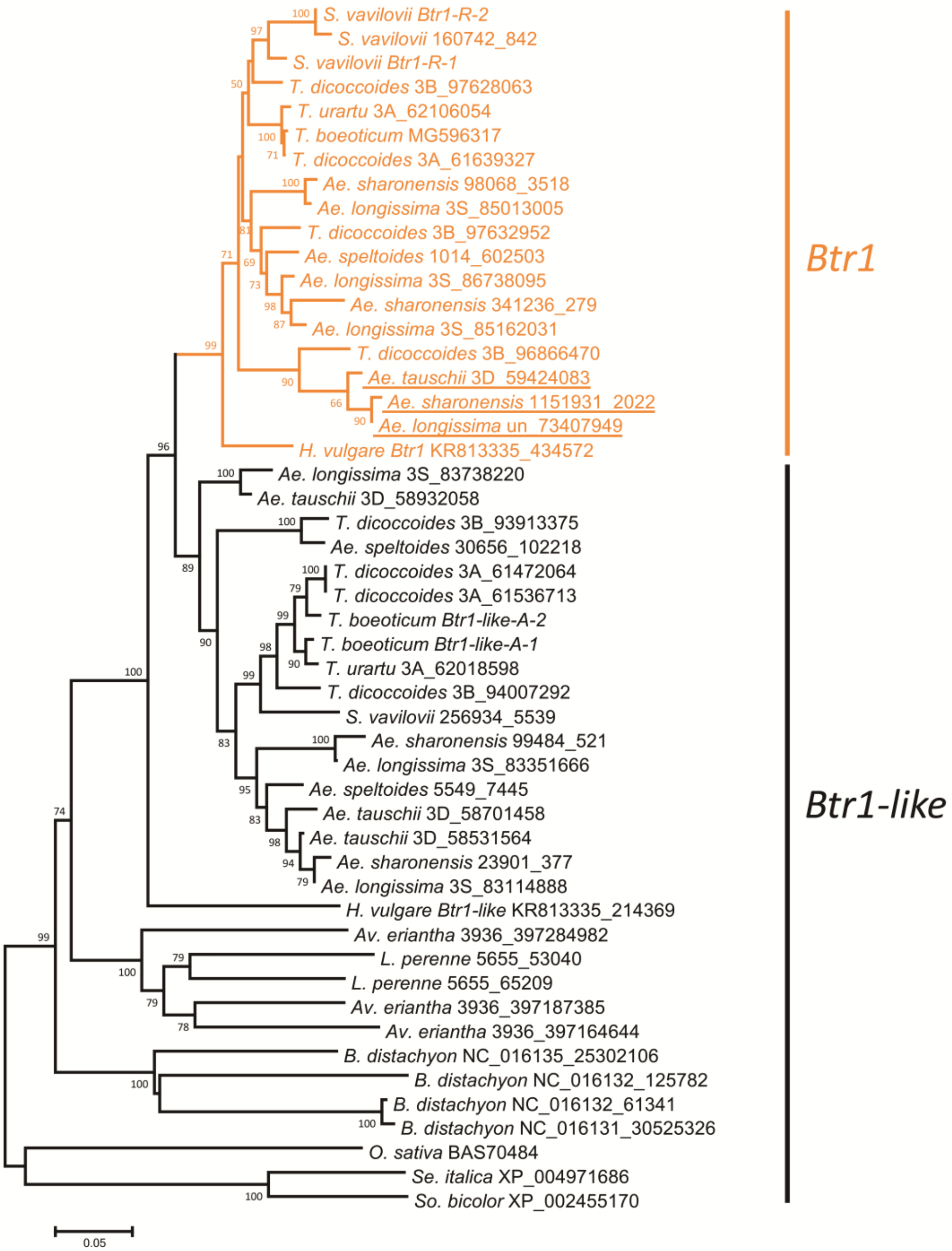

Fig. S1. Phylogenetic analysis of Poaceae *Btr1* and *Btr1-like* genes including those likely non-translated. Members of the *Btr1* clade are colored orange. The truncated *Btr1* copies in *Ae. tauschii* (chromosome 3D: 59424083-59424248) and *Ae. longissima* (un: 73407949-73408115) are shown underlined. Numbers given at a branch node indicate bootstrap probabilities (only those >50% are shown). The number refer to the accession / scaffold / contig and the start location of each gene is given in Table S3.

```
T_boeoticum_Btr1-like-A-1      ATGGCGCAGCCGCCGCCATGGAAGGCGATGTACCTGCCTGTGACGAGCGACGCGATCCGA 60
T_boeoticum_Btr1-like-A-2      ATGGCGCAGCCGCCGCCATGGAGGACGATGTACCTGTCTGTGACGAGCGAGGCGATCCGA 60
                               ********************** * *********** ************* *********

T_boeoticum_Btr1-like-A-1      TCCGCCGCCGCCGTCAAGCGAAGCGTCGCCGCCGCGCGTACCGAGCTGGCGTCCCCGCAG 120
T_boeoticum_Btr1-like-A-2      TCCGCCGCCCGCGTCAAGCAAAGCGTCGCCGCCGCGCGTCGCGATCTGGCGTCCCCGC-- 118
                               *********  ******** *******************  *** *************

T_boeoticum_Btr1-like-A-1      GTGGTGCTGGACACCCGCGACGCCGAGGGGCGGTACACCTTGCTGGAGTCCGCGCTGACC 180
T_boeoticum_Btr1-like-A-2      -TGGTGCTGGACACCCGCGACTCCGAGGGGCGGTACACCTTGCTGGAGTCCGCGCTGACA 177
                                ******************** *************************************

T_boeoticum_Btr1-like-A-1      CACATCGACCACGCATCCGGCTCCCTCTCCGCTTTCATCATCAACATGGTGGTGGCCGAG 240
T_boeoticum_Btr1-like-A-2      CACATCGACCACGCATCCGGCTCCCTCTCCGCTTTCATCATCAACACGGTGGTGGCCGAG 237
                               ********************************************** *************

T_boeoticum_Btr1-like-A-1      CGCCTGACGCTCCACGGCTGCGGCGCCGTCCCGTCGGAGCCGGTGGCCCGCGTCGGCGAC 300
T_boeoticum_Btr1-like-A-2      CGCCTGACGCTCCACGGCTGCGGCGCCGTCCCATCGGAGCCGGTGGCCCGCATCGGCGAC 297
                               ******************************** ****************** ********

T_boeoticum_Btr1-like-A-1      CTCCGCGACGGCCACGGGCGCCACGACGAGTGGCTTGCTCTGATCAGGCTCCAGGCCGCC 360
T_boeoticum_Btr1-like-A-2      CTCCGCGACGGCCACGGGCGCCACGACGAGTGGCTTGCTCTCGTCAGGCTCCAGGCCGCC 357
                               *****************************************  *****************

T_boeoticum_Btr1-like-A-1      AGGGAGCACGCCCAGGACGCGCTCCGCCGAGTGGAGGGGGCCTACACCCTCCTGGCCACC 420
T_boeoticum_Btr1-like-A-2      AGGGAGCACGCCGAGGACGCGCTCCGCCGAGTGGAGGGGGCCTACACCCTCCTGGGCTCC 417
                               ************ ****************************************** * **

T_boeoticum_Btr1-like-A-1      GTCGGGTTCATGCTTCACAGCCAGAACCCCGACGCTCCAGGGCGCCGGCAAGCCATGGAA 480
T_boeoticum_Btr1-like-A-2      GTCCGGTTCATGCTTCACAGCCAGAACCCCGACGCTCCAGGGCGCCGGCAAGCCATGGAA 477
                               *** ********************************************************

T_boeoticum_Btr1-like-A-1      GGGCAGCTCCACGCCCTCGACCTCCAGCCCGTGGTGGTCGGCGTGGCGAGCATGTCCGCG 540
T_boeoticum_Btr1-like-A-2      GGGCAGCTCCACGCCCTCGATCTCCAGCCCGTGGTGGTCGGCGTGGCGAGCATGTCCGCG 537
                               ******************** ***************************************

T_boeoticum_Btr1-like-A-1      CTGGCCTCCTTGGCCACCCAGCCCCCCATCCGCTACCGCATCCAGTGA 588
T_boeoticum_Btr1-like-A-2      CTGGCCTCCTTGGCCACCCAGCCCCCCATCCGCTACCGCATCCAGTGA 585
                               ************************************************
```

a

```
T_boeoticum_BTR1-LIKE-A-1      MAQPPPWKAMYLPVTSDAIRSAAAVKRSVAAARTELASPQVVLDTRDAEGRYTLLESALT 60
T_boeoticum_BTR1-LIKE-A-2      MAQPPPWRTMYLSVTSEAIRSAARVKQSVAAARRDLASP-LVLDTRDSEGRYTLLESALT 59
                               *******::***.***:****** **:****** :**** :******:************

T_boeoticum_BTR1-LIKE-A-1      HIDHASGSLSAFIINMVVAERLTLHGCGAVPSEPVARVGDLRDGHGRHDEWLALIRLQAA 120
T_boeoticum_BTR1-LIKE-A-2      HIDHASGSLSAFIINTVVAERLTLHGCGAVPSEPVARIGDLRDGHGRHDEWLALVRLQAA 119
                               *************** *********************:****************:*****

T_boeoticum_BTR1-LIKE-A-1      REHAQDALRRVEGAYTLLATVGFMLHSQNPDAPGRRQAMEGQLHALDLQPVVVGVASMSA 180
T_boeoticum_BTR1-LIKE-A-2      REHAEDALRRVEGAYTLLGSVRFMLHSQNPDAPGRRQAMEGQLHALDLQPVVVGVASMSA 179
                               ****:*************.:* **************************************

T_boeoticum_BTR1-LIKE-A-1      LASLATQPPIRYRIQ 195
T_boeoticum_BTR1-LIKE-A-2      LASLATQPPIRYRIQ 194
                               ***************
```

b

Fig. S2. Alignment of the products of the *T. boeoticum* genes *Btr1-like-A-1* and *Btr1-like-A-2*.

```
H_vulgare_Btr1_KR813335:434572-435162     ATGGCGCAGCCGCCGCAATGGAAGGCGATGTACCAGTATGTCGCGCGACGGGCGCACGAC 60
S_vavilovii_Btr1-R-1                      ATGGCGCAGCCGCCGCAATGGAAGGCGATGTACCAGTACGTGGCGATACGGGCGCACGAC 60
S_vavilovii_contig_160742:842-1852        ATGGCGCAGCCACCGCAATGGAAGGCGATGTACCAGTACGTGGCGATACGGGCGCACGAC 60
S_vavilovii_Btr1-R-2                      ATGGCGCAGCCACCGCAATGGAAGGCGATGTACCAGTACGTGGCGATACGGGCGCACGAC 60
                                          *********** ************************** ** ***  *************

H_vulgare_Btr1_KR813335:434572-435162     GGCTGCGCCCGCGTCGAGGAAAGCGTCGCCGCGGCGCGCGGAGCGCTGGCGACCCCGATG 120
S_vavilovii_Btr1-R-1                      GGCTGCGCCCGCGTCCAGGAAAGTGTCGCCGACGCGCGCAGGGCGCTGGCGTCACCGCTG 120
S_vavilovii_contig_160742:842-1852        GGCTGCGCCCGCGTCCAGGAAAGTGTCGCCGACGCGCGCAGGGCGCTGGCGTCACCGCTG 120
S_vavilovii_Btr1-R-2                      GGCTGCGCCCGCGTCGAGGAAAGTATCGCCGACGCGCGCAGGGCGCTGGCGTCCCCGCTG 120
                                          *************** *******  ******  ****** * ********* * *** **

H_vulgare_Btr1_KR813335:434572-435162     GTGCTGGACACCCGCGACGCCGCGGGGCGGTGCACGTTGCTGCATTCCGCGGTGACCCAC 180
S_vavilovii_Btr1-R-1                      GTGCTGGACACCCGCGACGCCGCGGGGCGGTACACCTCCTTGCACTCCGCGATGACCCAC 180
S_vavilovii_contig_160742:842-1852        GTGCTGGACACCCGCGACGCCGCGGGGCGGTACACCTCGTTGCACTCCGCGATGACCCAC 180
S_vavilovii_Btr1-R-2                      GTGCTGGACACCCGCGACGCCGCGGGGCGGTACACCTCGTTGCACTCCGCGATGACCCAC 180
                                          ******************************* *** *   **** ****** ********

H_vulgare_Btr1_KR813335:434572-435162     GTCGAGCACGCATCCGACTGCCTCTCCGGTTTCATAGTCAGCGTGGTGGTGGCGGAGCTC 240
S_vavilovii_Btr1-R-1                      GTCGAGCACGCATCCGGCTGCCTCTCCGGTGTCATATTCAGCATGGTGGTGGCCGAGCTC 240
S_vavilovii_contig_160742:842-1852        GTCGAGCACGCATCCGGCTACCTATCCGGTGTCATATTCAGCATGGTGGTGGCCGAGCTC 240
S_vavilovii_Btr1-R-2                      GTCGAGCACGCATCCGGCTACCTATCCGGTGTCATATTCAGCATGGTGGTGGCCGAGCTC 240
                                          **************** ** *** ****** ***** ***** ********** ******

H_vulgare_Btr1_KR813335:434572-435162     CTGGTGCTCCATGGCTGCGGGGCCGTCCCTTCGAGGCCGGTGGCCAGCATCGGCGGCCTC 300
S_vavilovii_Btr1-R-1                      CTGGCGCTCCATGGCTGCGGGGCCGTCCCGTCGAGGCCGGTGGCCGGCATCGGCGACCTC 300
S_vavilovii_contig_160742:842-1852        CTGGCGCTCCATGGCTGCGGGGCCGTCCCGTCGAGACCGGTGGCCGGCATCGGCGACCTC 300
S_vavilovii_Btr1-R-2                      CTGGCGCTCCATGGCTGCGGGGCCGTCCCGTCGAGACCGGTGGCCGGCATCGGCGACCTC 300
                                          **** ************************ ***** ********* ********* ****

H_vulgare_Btr1_KR813335:434572-435162     CGCCGCAACCGCGACGACCACGACGAGTGGCTCGCTCTGAGCAGGCTCGAGGCCGCCAGG 360
S_vavilovii_Btr1-R-1                      CGCCGCGACCGCGACGACCACGACGAGTGGCTCGCTCTGAGCAGGCTCGAGGCCGCCAGG 360
S_vavilovii_contig_160742:842-1852        CGCCGCGACCGCGACGACCACGACGAGTGGCTGGCTGTGAGCAGGCTCGAGGCCGCCAGG 360
S_vavilovii_Btr1-R-2                      CGCCGCGACCGCGACGACCACGACGAGTGGCTGGCTGTGAGCAGGCTCGAGGCCGCCAGG 360
                                          ****** ************************* *** ***********************

H_vulgare_Btr1_KR813335:434572-435162     GAGCACGGCCAGGACGCGCTCCGCGGAGTGGAGGGGGCCTTCACCCTCCTGGCCTCCGTC 420
S_vavilovii_Btr1-R-1                      GAGCACGCCCAGGATGCGCTCCGCGGGGTGGAGGGCGCCTTCACCCTCCTGGCCTCCGTG 420
S_vavilovii_contig_160742:842-1852        GAGCACGCCCAGGACGCGCTCCGCGGGGTGGAGGGCGCCTTCACCCTCCTGGCCTCCGTC 420
S_vavilovii_Btr1-R-2                      GAGCACGCCCAGGACGCGCTCCGCGGGGTGGAGGGCGCCTTCACCCTCCTGGCCTCCGTC 420
                                          ******* ****** *********** ******** *********************** 

H_vulgare_Btr1_KR813335:434572-435162     CGGTTCATGCTTCGCAGCCGGACCCCTGACGCCGCCGGGCGCCGGCAAGCCATGGAAGAG 480
S_vavilovii_Btr1-R-1                      CGGTTCATGCTTCACAGCCGGACCCCCGACGCTGCCGGGCGCCGCCAAGCCATGGAAGAG 480
S_vavilovii_contig_160742:842-1852        CGGTTCATGCTTCACAGCCGTACCCCTGACGCTGCTGGGCGCCGGCAAGCCATGGAAGAG 480
S_vavilovii_Btr1-R-2                      CGGTTCATGCTTCACAGCCGTACCCCTGACGCTGCTGGGCGCCGGCAAGCCATGGAAGAG 480
                                          ************* ****** ***** ***** ** ******** ***************

H_vulgare_Btr1_KR813335:434572-435162     CAGCTCCACGCCGCCGCCGTCGAACTTCAGGCCGTGGTGGGCAGCGTGGCGAACATGTCC 540
S_vavilovii_Btr1-R-1                      CAGCTCCACGCCGCCGCCGTCGAACTCCAGGCCGTGGTGGGCAGCGTGGCCAACTTGTCC 540
S_vavilovii_contig_160742:842-1852        CAGCTCCACGCCGCCGCCGTCGAACTCCAGGCCGTGGTGGGCAGCGTGGCC----TGTCC 536
S_vavilovii_Btr1-R-2                      CAGCTCCACGCCGCCGCCGTCGAACTCCAGGCCGTGGTGGGCAGCGTGGCC----TGTCC 536
                                          ************************** ***********************     *****

H_vulgare_Btr1_KR813335:434572-435162     GCGCTGGCTTTCTTGGCCACTCAGCCTGCCATCCGCAACCGCATCCAGTGA--------- 591
S_vavilovii_Btr1-R-1                      GCGCTGGCCTTCTTGGCCACCCAGCCTGCCATCCGCAACCGCTTCCAGTGA--------- 591
S_vavilovii_contig_160742:842-1852        GCGCTGGCCTTCCTGGCCACCCAGCCTGCCATCCGCAACCGAATCCAGTGAGAGGTCCCT 596
S_vavilovii_Btr1-R-2                      GCGCTGGCCTTCCTGGCCACCCAGCCTGCCATCCGCAACCGAATCCAGTGA--------- 587
                                          ******** *** ******* ********************  ********

H_vulgare_Btr1_KR813335:434572-435162     ------------------------------------------------------------ 591
S_vavilovii_Btr1-R-1                      ------------------------------------------------------------ 591
S_vavilovii_contig_160742:842-1852        TGACTTGCTCTGTCTGCACGATCTGCTTTGCTTGATATCGATGGTTCGCGTTGGCTAGGG 656
S_vavilovii_Btr1-R-2                      ------------------------------------------------------------ 587

H_vulgare_Btr1_KR813335:434572-435162     ------------------------------------------------------------ 591
S_vavilovii_Btr1-R-1                      ------------------------------------------------------------ 591
S_vavilovii_contig_160742:842-1852        CTAGCCCATGCATGGTCTATGTGTCCGTCGAATTCGAACTACGTACTGTTTTCGGTTCTG 716
S_vavilovii_Btr1-R-2                      ------------------------------------------------------------ 587

H_vulgare_Btr1_KR813335:434572-435162     ------------------------------------------------------------ 591
S_vavilovii_Btr1-R-1                      ------------------------------------------------------------ 591
S_vavilovii_contig_160742:842-1852        TTGTTGTTAACTGCAAGGATCTCTATATACCGGGGACCTAATTAGATGTATACTTTGATG 776
S_vavilovii_Btr1-R-2                      ------------------------------------------------------------ 587
```

```
H_vulgare_Btr1_KR813335:434572-435162      ------------------------------------------------------------ 591
S_vavilovii_Btr1-R-1                       ------------------------------------------------------------ 591
S_vavilovii_contig_160742:842-1852         TTTGTCGATCTGAAATCCGAGAGATATTTGTTGGTAATTTGGCGTGGTTCATCACTTAAT 836
S_vavilovii_Btr1-R-2                       ------------------------------------------------------------ 587

H_vulgare_Btr1_KR813335:434572-435162      ------------------------------------------------------------ 591
S_vavilovii_Btr1-R-1                       ------------------------------------------------------------ 591
S_vavilovii_contig_160742:842-1852         TACTGTCATGCATGATTGTATGATGATGATGCGTCGATCATGCATGCCTGGGTGGGTTCG 896
S_vavilovii_Btr1-R-2                       ------------------------------------------------------------ 587

H_vulgare_Btr1_KR813335:434572-435162      ------------------------------------------------------------ 591
S_vavilovii_Btr1-R-1                       ------------------------------------------------------------ 591
S_vavilovii_contig_160742:842-1852         TACGTTAGTTGCAACTTGCAACGTACATTGGCAGTGCTGTTTTCCTTTGTGGTGGCATTG 956
S_vavilovii_Btr1-R-2                       ------------------------------------------------------------ 587

H_vulgare_Btr1_KR813335:434572-435162      ------------------------------------------------------- 591
S_vavilovii_Btr1-R-1                       ------------------------------------------------------- 591
S_vavilovii_contig_160742:842-1852         CACTTTGCAGTTGAGATTCAACTATGATCGATCAGCGAGCGATATCGTAGAATAA 1011
S_vavilovii_Btr1-R-2                       ------------------------------------------------------- 587
```

a

```
H_vulgare_BTR1_KR813335:434572-435162      MAQPPQWKAMYQYVARRAHDGCARVEESVAAARGALATPMVLDTRDAAGRCTLLHSAVTH 60
S_vavilovii_BTR1-R-1                       MAQPPQWKAMYQYVAIRAHDGCARVEESIADARRALASPLVLDTRDAAGRYTSLHSAMTH 60
S_vavilovii_contig_160742:842-1852         MAQPPQWKAMYQYVAIRAHDGCARVQESVADARRALASPLVLDTRDAAGRYTSLHSAMTH 60
                                           *************** *********:**:* ** ***:*:********** * ****:**
H_vulgare_BTR1_KR813335:434572-435162      VEHASDCLSGFIVSVVVAELLVLHGCGAVPSRPVASIGGLRRNRDDHDEWLALSRLEAAR 120
S_vavilovii_BTR1-R-1                       VEHASGCLSGVIFSMVVAELLALHGCGAVPSRPVAGIGDLRRDRDDHDEWLALSRLEAAR 120
S_vavilovii_contig_160742:842-1852         VEHASGYLSGVIFSMVVAELLALHGCGAVPSRPVAGIGDLRRDRDDHDEWLAVSRLEAAR 120
                                           *****. ***.*.*:******.*************.**.***:*********:*******
H_vulgare_BTR1_KR813335:434572-435162      EHGQDALRGVEGAFTLLASVRFMLRSRTPDAAGRRQAMEEQLHAAAVELQAVVGSVANMS 180
S_vavilovii_BTR1-R-1                       EHAQDALRGVEGAFTLLASVRFMLHSRTPDAAGRRQAMEEQLHAAAVELQAVVGSVANLS 180
S_vavilovii_contig_160742:842-1852         EHAQDALRGVEGAFTLLASVRFMLHSRTPDAAGRRQAMEEQLHAAAVELQAVL------R 174
                                           **.*******************:***************************:
H_vulgare_BTR1_KR813335:434572-435162      ALAFLATQPAIRNRIQ 196
S_vavilovii_BTR1-R-1                       ALAFLATQPAIRNRFQ 196
S_vavilovii_contig_160742:842-1852         F-NYDRSASDIVE--- 186
                                           :  : . * :
```

b

Fig. S3. The *Btr1* genes present in *S. vavilovii.* (a) Nucleotide alignment between S_vavilovii_contig_160742, *Btr1-R-1, Btr1-R-2* and barley *Btr1*. Exons in S_vavilovii_contig_160742 and *Btr1-R-2* are shown boxed. (b) Polypeptide alignment between the products of the *S. vavilovii Btr1* genes and barley *Btr1*.

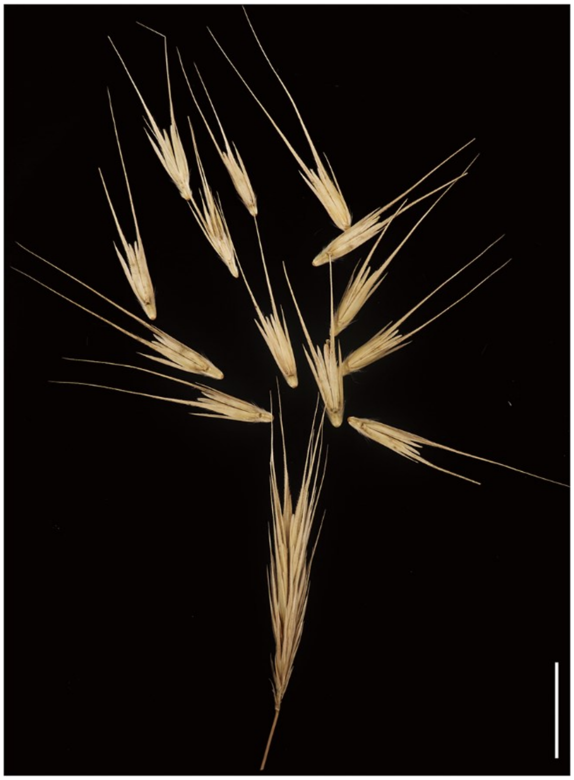

Fig. S4. The mature spike of *S. vavilovii.* Scale bar, 2cm.

```
H_vulgare_Btr1_KR813335:434572-435162    ATGGCGCAGCCGCCGCAATGGAAGGCGATGTACCAGTATGTCGCGCGACGGGCGCACGAC 60
Ae_sharonensis_contig_98068:3458-2918    ATGGCGCAGCCGCCGCAGTGGAAGGCGATGTACCAGTATGTGGCGATACGGGCGCACGAC 60
Ae_sharonensis_contig_341236:279-739     ATGGCGCAGCCGCCGCAGTGGAAGGCGATGTACCAGTATGTGGC---ACGGAAGAGATGG 57
                                         ***************** *********************** **   ****  *

H_vulgare_Btr1_KR813335:434572-435162    GGCTGCGCCCGCGTCGAGGAAAGCGTCGCCGCGGCGCGCGGAGCGCTGGCGACCCCGATG 120
Ae_sharonensis_contig_98068:3458-2918    AGCTGCGCCCGAGTGGAGGAAAGTGTCGCTGCCGCGCGTAGGGAGCTGGCGTCCCCGCGG 120
Ae_sharonensis_contig_341236:279-739     CGCTGCACTCGCGTTGAGGAAAGTGTCGCTGCCGCGCGTAGGGTGCTGGCGTCCTCGCAG 117
                                          ***** * ** ** ******** ***** ** *****  * * ******* ** **  *

H_vulgare_Btr1_KR813335:434572-435162    GTGCTGGACACCCGCGACGCCGCGGGGCGGTGCACGTTGCTGCATTCCGCGGTGACCCAC 180
Ae_sharonensis_contig_98068:3458-2918    GTGCTGGACACCCGCAACGCCGCGGGGCGGTATGCCTTGTTGCATTCCGCGATGACCCAC 180
Ae_sharonensis_contig_341236:279-739     CTGCTGGACACCCGTGACGCCGCGGGGCGGTACACCTTGTTGCATTCCCCG--------- 168
                                          *************  ***************   * *** ******** **

H_vulgare_Btr1_KR813335:434572-435162    GTCGAGCACGCATCCGACTGCCTCTCCGGTTTCATAGTCAGCGTGGTGGTGGCGGAGCTC 240
Ae_sharonensis_contig_98068:3458-2918    GTCGAGCACGCATCCGGCTGCCTCTCCGGTGTCATATTCAGCATGGGGGTGGCCGAGCAA 240
Ae_sharonensis_contig_341236:279-739     ------------------------------------------------------------ 168

H_vulgare_Btr1_KR813335:434572-435162    CTGGTGCTCCATGGCTGCGGGGCCGTCCCTTCGAGGCCGGTGGCCAGCATCGGCGGCCTC 300
Ae_sharonensis_contig_98068:3458-2918    CTGGCGCTCTATGGCTGCGGCGCCGTTCCGTCGAGGCCGGTAGCCGGCATCGGCGACCTC 300
Ae_sharonensis_contig_341236:279-739     ------------------------------------------------------------ 168

H_vulgare_Btr1_KR813335:434572-435162    CGCCGCAACCGCGACGACCACGACGAGTGGCTCGCTCTGAGCAGGCTCGAGGCCGCCAGG 360
Ae_sharonensis_contig_98068:3458-2918    CGCCGCGACCGCGGCGACCACGATGAGTGGCTCGCTCTGAGCAGGCTCGAGGCCGCCAGG 360
Ae_sharonensis_contig_341236:279-739     -------ACCGCGACGACCACAACGAGTGGCTCGCTCTGAGCAGGCTCGAGGCCGCCAGG 221
                                                ****** ******* * ************************************

H_vulgare_Btr1_KR813335:434572-435162    GAGCACGGCCAGGACGCGCTCCGCGGAGTGGAGGGGGCCTTCACCCTCCTGGCCTCCGTC 420
Ae_sharonensis_contig_98068:3458-2918    GAGCACGCCCAGGACGCGCTCCCCGGGGTGGAGGGGGCTTTCACCCTGCTGGCCTCCGTC 420
Ae_sharonensis_contig_341236:279-739     GAGCACGCCCAGGACGCCCTCCGCGGGGTGGAGAGGGCCTTCACCCTCCTGGCATCCGTC 281
                                         ******* ********* **** *** ****** **** ******** ***** ******

H_vulgare_Btr1_KR813335:434572-435162    CGGTTCATGCTTCGCAGCCGGACCCCTGACGCCGCCGGGCGCCGGCAAGCCATGGAAGAG 480
Ae_sharonensis_contig_98068:3458-2918    CGGTTCCTGCTTCACAGCCGGAGCCCTGACGCTGCCGGCCGCCGGCAAGCCATCGAAGAG 480
Ae_sharonensis_contig_341236:279-739     CGGTTCCTGCTTCACAGCCGGACCCCCGACGCTGCCGGGCGCCGGCAAGCCATGGAAGAG 341
                                         ****** ****** ******** *** ***** ***** ************** ******

H_vulgare_Btr1_KR813335:434572-435162    CAGCTCCACGCCGCCGCCGTCGAACTTCAGGCCGTGGTGGGCAGCGTGGCGAACATGTCC 540
Ae_sharonensis_contig_98068:3458-2918    CAGCTCCACGCCGCCACCGACGAACTCCAGGCCGTCGTGGGCAGCGTGGCCAACATGTCC 540
Ae_sharonensis_contig_341236:279-739     CAGCTCCACGCGGCCGGCGTCGAACTCCAAGCCGCGGTAGGCAGCGTGGCCAACATGTCC 401
                                         *********** ***  ** ****** ** ****  ** *********** *********

H_vulgare_Btr1_KR813335:434572-435162    GCGCTGGCTTTCTTGGCCACTCAGCCTGCCATCCGCAACCGCATCCAGTGA 591
Ae_sharonensis_contig_98068:3458-2918    GCGCTGGCCTTCTTGGCCACCCAGCCTGCCATCCGCAACCGTATCCAGTGA 591
Ae_sharonensis_contig_341236:279-739     GCGCTGGCCTTCATGGCCACCCAGCCTGCCATCCGCAACCGCATCCAGTGA 452
                                         ******** *** ******* ******************** *********
```

a

```
H_vulgare_BTR1_KR813335:434572-435162    MAQPPQWKAMYQYVARRAHDGCARVEESVAAARGALATPMVLDTRDAAGRCTLLHSAVTH 60
Ae_sharonensis_contig_98068:3458-2918    MAQPPQWKAMYQYVAIRAHDSCARVEESVAAARRELASPRVLDTRNAAGRYALLHSAMTH 60
Ae_sharonensis_contig_341236:279-739     MAQPPQWKAMYQYVA-RKRWRCTRVEESVAAARRVLASSQLLDTRDAAG----------- 48
                                         *************** * :  *:**********  **:. :****:***

H_vulgare_BTR1_KR813335:434572-435162    VEHASDCLSGFIVSVVVAELLVLHGCGAVPSRPVASIGGLRRNRDDHDEWLALSRLEAAR 120
Ae_sharonensis_contig_98068:3458-2918    VEHASGCLSGVIFSMGVAEQLALYGCGAVPSRPVAGIGDLRRDRGDHDEWLALSRLEAAR 120
Ae_sharonensis_contig_341236:279-739     ------------------------------------------------------RLEAAR 54
                                                                                               ******

H_vulgare_BTR1_KR813335:434572-435162    EHGQDALRGVEGAFTLLASVRFMLRSRTPDAAGRRQAMEEQLHAAAVELQAVVGSVANMS 180
Ae_sharonensis_contig_98068:3458-2918    EHAQDALPGVEGAFTLLASVRFLLHSRSPDAAGRRQAIEEQLHAATDELQAVVGSVANMS 180
Ae_sharonensis_contig_341236:279-739     EHAQDALRGVERAFTLLASVRFLLHSRTPDAAGRRQAMEEQLHAAGVELQAAVGSVANMS 114
                                         **.**** *** **********:*:**:*********:*******  ****.********

H_vulgare_BTR1_KR813335:434572-435162    ALAFLATQPAIRNRIQ 196
Ae_sharonensis_contig_98068:3458-2918    ALAFLATQPAIRNRIQ 196
Ae_sharonensis_contig_341236:279-739     ALAFMATQPAIRNRIQ 130
                                         ****:***********
```

b

Fig. S5. The *Btr1* genes present in *Ae. sharonensis* (a) Nucleotide alignment between Ae_sharonensis_contig_98068, Ae_sharonensis_contig_341236 and barley *Btr1*. Exons in Ae_sharonensis_contig_341236 are shown boxed. (b) Polypeptide alignment between the products of the *Ae. sharonensis Btr1* genes and barley *Btr1*.

```
H_vulgare_Btr1_KR813335:434572-435162      ATGGCGCAGCCGCCGCAATGGAAGGCGATGTACCAGTATGTCGCGCGACGGGCGCACGAC 60
Ae_sharonensis_98068:3518-2928             ATGGCGCAGCCGCCGCAGTGGAAGGCGATGTACCAGTATGTGGCGATACGGGCGCACGAC 60
Ae_longissima_3S:86738095-86738685         ATGGCGCAGCCGCCGCAGTGGAAGGCGATGTACCAGTATGTGGCGATACGAGCGCACGAC 60
Ae_sharonensis_1151931:2022-1843           ATGGCGCAGCCGCCGCAATGCAAGGCGATGTACGTGTATGTGGCGAGACGGGCGCGCGAA 60
Ae_longissima_un:73407949-73408128         ATGGCGCAGCCGCCGCAATGCAAGGCGATGTACGTGTATGTGGCGAGACGGGCGCGCGAA 60
Ae_tauschii_3D:59424083-59424261           A-GGCGCAGCCGCCGCAATGGAAGGCGATGTACGTGTATGTGGCGCGACGGGCGCGCGAA 59
                                           * *************** ** ************  ****** ***  *** **** ***

H_vulgare_Btr1_KR813335:434572-435162      GGCTGCGCCCGCGTCGAGGAAAGCGTCGCCGCGGCGCGCGGAGCGCTGGCGACCCCGATG 120
Ae_sharonensis_98068:3518-2928             AGCTGCGCCCGAGTGGAGGAAAGTGTCGCTGCCGCGCGTAGGGAGCTGGCGTCCCCGCGG 120
Ae_longissima_3S:86738095-86738685         GGCTGCACCCGCGTTGAGGAAAGTGTCGCCGCCGCGCGTAGGGTGCTGGCGTCCCCGGAG 120
Ae_sharonensis_1151931:2022-1843           GGCTGCTCTCGCGTCGAGCAAAGTGTCGCGGCGGCGCGTACGGTGCTGGCGTCCCCGCTG 120
Ae_longissima_un:73407949-73408128         GGCTGCTCTCGCGTCGAGCAAAGTGTCGCGGCGGCGCGTACGGTGCTGGCGTCCCCGCTG 120
Ae_tauschii_3D:59424083-59424261           GGCTGCTCTCGCGTCGAGCACAGTGTCGCGGCCGCGCGTACGGTGCTGGCGTCCCCGCTG 119
                                            ***** * ** ** *** * ** ***** ** *****     * ******* *****  *

H_vulgare_Btr1_KR813335:434572-435162      GTGCTGGACACCCGCGACGCCGCGGGGCGGTGCACGTTGCTGCATTCCGCGGTGACCCAC 180
Ae_sharonensis_98068:3518-2928             GTGCTGGACACCCGCAACGCCGCGGGGCGGTATGCCTTGTTGCATTCCGCGATGACCCAC 180
Ae_longissima_3S:86738095-86738685         TTGCTGGACACCCGCGACGCCGCGGGGCGGTACACCTTGTTGCATTCCGCGATGACCCAC 180
Ae_sharonensis_1151931:2022-1843           GTGCTGGACACCCGCAACGCCGCGGGCCGGTACACCTTGTTGCATTCTACTTGGATATGA 180
Ae_longissima_un:73407949-73408128         GTGCTGGACACCCGCAACGCCGCGGGCCGGTACACCTTGTTGCATTCTACTTGGATATGA 180
Ae_tauschii_3D:59424083-59424261           GTGCTGGACACCCGCAACGCCGCGGGCCGGTACACCTTGTTGCATTCTACTTGGATATGA 179
                                            ************** ********** ****    * *** *******  *   **

H_vulgare_Btr1_KR813335:434572-435162      GTCGAGCACGCATCCGACTGCCTCTCCGGTTTCATAGTCAGCGTGGTGGTGGCGGAGCTC 240
Ae_sharonensis_98068:3518-2928             GTCGAGCACGCATCCGGCTGCCTCTCCGGTGTCATATTCAGCATGGGGGTGGCCGAGCAA 240
Ae_longissima_3S:86738095-86738685         GTCGAGCACGCATCCGGCTGCCTCTCCGGTGGCGTATTCAGCATGGGGGTGGCCGAGCTC 240
Ae_sharonensis_1151931:2022-1843           ------------------------------------------------------------ 180
Ae_longissima_un:73407949-73408128         ------------------------------------------------------------ 180
Ae_tauschii_3D:59424083-59424261           ------------------------------------------------------------ 179

H_vulgare_Btr1_KR813335:434572-435162      CTGGTGCTCCATGGCTGCGGGGCCGTCCCTTCGAGGCCGGTGGCCAGCATCGGCGGCCTC 300
Ae_sharonensis_98068:3518-2928             CTGGCGCTCTATGGCTGCGGCGCCGTTCCGTCGAGGCCGGTAGCCGGCATCGGCGACCTC 300
Ae_longissima_3S:86738095-86738685         CTGGCGCTCCATGGCTGCGGCGCCGTTCCGTCGAGGCCGGTAGCCGGCATCGGCGACCTC 300
Ae_sharonensis_1151931:2022-1843           ------------------------------------------------------------ 180
Ae_longissima_un:73407949-73408128         ------------------------------------------------------------ 180
Ae_tauschii_3D:59424083-59424261           ------------------------------------------------------------ 179

H_vulgare_Btr1_KR813335:434572-435162      CGCCGCAACCGCGACGACCACGACGAGTGGCTCGCTCTGAGCAGGCTCGAGGCCGCCAGG 360
Ae_sharonensis_98068:3518-2928             CGCCGCGACCGCGGCGACCACGATGAGTGGCTCGCTCTGAGCAGGCTCGAGGCCGCCAGG 360
Ae_longissima_3S:86738095-86738685         CGCCGCGACCGCGACGACCACGACGAGTGGCTCGCTCTGAGCAGGCTCGAGGCCGCCAGG 360
Ae_sharonensis_1151931:2022-1843           ------------------------------------------------------------ 180
Ae_longissima_un:73407949-73408128         ------------------------------------------------------------ 180
Ae_tauschii_3D:59424083-59424261           ------------------------------------------------------------ 179

H_vulgare_Btr1_KR813335:434572-435162      GAGCACGGCCAGGACGCGCTCCGCGGAGTGGAGGGGGCCTTCACCCTCCTGGCCTCCGTC 420
Ae_sharonensis_98068:3518-2928             GAGCACGCCCAGGACGCGCTCCCCGGGGTGGAGGGGGCTTTCACCCTGCTGGCCTCCGTC 420
Ae_longissima_3S:86738095-86738685         GAGCACGCCCAGGACGCGCTCCGCGGGGTGGAGAGGGCCTTCACCCTCCTGGCATCCGTC 420
Ae_sharonensis_1151931:2022-1843           ------------------------------------------------------------ 180
Ae_longissima_un:73407949-73408128         ------------------------------------------------------------ 180
Ae_tauschii_3D:59424083-59424261           ------------------------------------------------------------ 179

H_vulgare_Btr1_KR813335:434572-435162      CGGTTCATGCTTCGCAGCCGGACCCCTGACGCCGCCGGGCGCCGGCAAGCCATGGAAGAG 480
Ae_sharonensis_98068:3518-2928             CGGTTCCTGCTTCACAGCCGGAGCCCTGACGCTGCCGGCCGCCGGCAAGCCATCGAAGAG 480
Ae_longissima_3S:86738095-86738685         CGGTTCCTGCTTCACAGCCGGACCCCCGACGCTGCCGGGCGCCGGCAAGCCATGGAAGAG 480
Ae_sharonensis_1151931:2022-1843           ------------------------------------------------------------ 180
Ae_longissima_un:73407949-73408128         ------------------------------------------------------------ 180
Ae_tauschii_3D:59424083-59424261           ------------------------------------------------------------ 179

H_vulgare_Btr1_KR813335:434572-435162      CAGCTCCACGCCGCCGCCGTCGAACTTCAGGCCGTGGTGGGCAGCGTGGCGAACATGTCC 540
Ae_sharonensis_98068:3518-2928             CAGCTCCACGCCGCCACCGACGAACTCCAGGCCGTCGTGGGCAGCGTGGCCAACATGTCC 540
Ae_longissima_3S:86738095-86738685         CAGCTCCACGCGGCCGGCGTCGAACTCCAGGCCGCGGTAGGCAGCGTGGCCAACATGTCC 540
Ae_sharonensis_1151931:2022-1843           ------------------------------------------------------------ 180
Ae_longissima_un:73407949-73408128         ------------------------------------------------------------ 180
Ae_tauschii_3D:59424083-59424261           ------------------------------------------------------------ 179

H_vulgare_Btr1_KR813335:434572-435162      GCGCTGGCTTTCTTGGCCACTCAGCCTGCCATCCGCAACCGCATCCAGTGA 591
Ae_sharonensis_98068:3518-2928             GCGCTGGCCTTCTTGGCCACCCAGCCTGCCATCCGCAACCGTATCCAGTGA 591
Ae_longissima_3S:86738095-86738685         GCGCTGGCCTTCATGGCCACCCAGCCTGCCATCCGCAACCGCATCCAGTGA 591
Ae_sharonensis_1151931:2022-1843           --------------------------------------------------- 180
Ae_longissima_un:73407949-73408128         --------------------------------------------------- 180
Ae_tauschii_3D:59424083-59424261           --------------------------------------------------- 179
```

a

```
H_vulgare_BTR1_KR813335:434572-435162      MAQPPQWKAMYQYVARRAHDGCARVEESVAAARGALATPMVLDTRDAAGRCTLLHSAVTH 60
Ae_sharonensis_98068:3518-2928             MAQPPQWKAMYQYVAIRAHDSCARVEESVAAARRELASPRVLDTRNAAGRYALLHSAMTH 60
Ae_longissima_3S:86738095-86738685         MAQPPQWKAMYQYVAIRAHDGCTRVEESVAAARRVLASPELLDTRDAAGRYTLLHSAMTH 60
Ae_sharonensis_1151931:2022-1843           MAQPPQCKAMYVYVARRAREGCSRVEQSVAAARTVLASPLVLDTRNAAGRYTLLHST--- 57
Ae_longissima_un:73407949-73408128         MAQPPQCKAMYVYVARRAREGCSRVEQSVAAARTVLASPLVLDTRNAAGRYTLLHST--- 57
                                           ****** **** *** **::.*:***:******  **:* :****:**** :****:

H_vulgare_BTR1_KR813335:434572-435162      VEHASDCLSGFIVSVVVAELLVLHGCGAVPSRPVASIGGLRRNRDDHDEWLALSRLEAAR 120
Ae_sharonensis_98068:3518-2928             VEHASGCLSGVIFSMGVAEQLALYGCGAVPSRPVAGIGDLRRDRGDHDEWLALSRLEAAR 120
Ae_longissima_3S:86738095-86738685         VEHASGCLSGGVFSMGVAELLALHGCGAVPSRPVAGIGDLRRDRDDHDEWLALSRLEAAR 120
Ae_sharonensis_1151931:2022-1843           -------------------------------------------------WI--------- 59
Ae_longissima_un:73407949-73408128         -------------------------------------------------WI--------- 59
                                                                                            *:

H_vulgare_BTR1_KR813335:434572-435162      EHGQDALRGVEGAFTLLASVRFMLRSRTPDAAGRRQAMEEQLHAAAVELQAVVGSVANMS 180
Ae_sharonensis_98068:3518-2928             EHAQDALPGVEGAFTLLASVRFLLHSRSPDAAGRRQAIEEQLHAATDELQAVVGSVANMS 180
Ae_longissima_3S:86738095-86738685         EHAQDALRGVERAFTLLASVRFLLHSRTPDAAGRRQAMEEQLHAAGVELQAAVGSVANMS 180
Ae_sharonensis_1151931:2022-1843           ------------------------------------------------------------ 59
Ae_longissima_un:73407949-73408128         ------------------------------------------------------------ 59

H_vulgare_BTR1_KR813335:434572-435162      ALAFLATQPAIRNRIQ 196
Ae_sharonensis_98068:3518-2928             ALAFLATQPAIRNRIQ 196
Ae_longissima_3S:86738095-86738685         ALAFMATQPAIRNRIQ 196
Ae_sharonensis_1151931:2022-1843           ---------------- 59
Ae_longissima_un:73407949-73408128         ---------------- 59
```

b

Fig. S6. The truncated *Btr1* genes present in *Ae. tauschii*, *Ae. longissima* and *Ae. sharonensis.* (a) Nucleotide alignment between the truncated *Ae. tauschii Btr1* (Ae_tauschii_3D: 59424083-59424261), *Ae. longissima Btr1* (Ae_longissima_3S: 86738095-86738685), a truncated *Ae. longissima Btr1* (Ae_longissima_un: 73407949-73408128), *Ae. sharonensis Btr1* (Ae_sharonensis_98068: 3518-2928), a truncated *Ae. sharonensis Btr1* (Ae_sharonensis_1151931: 2022-1843) and barley *Btr1*. The putative stop codon in the *Btr1* sequence is marked in bold. (b) Polypeptide alignment between the products of the *Aegilops* spp. *Btr1* genes and barley *Btr1*.

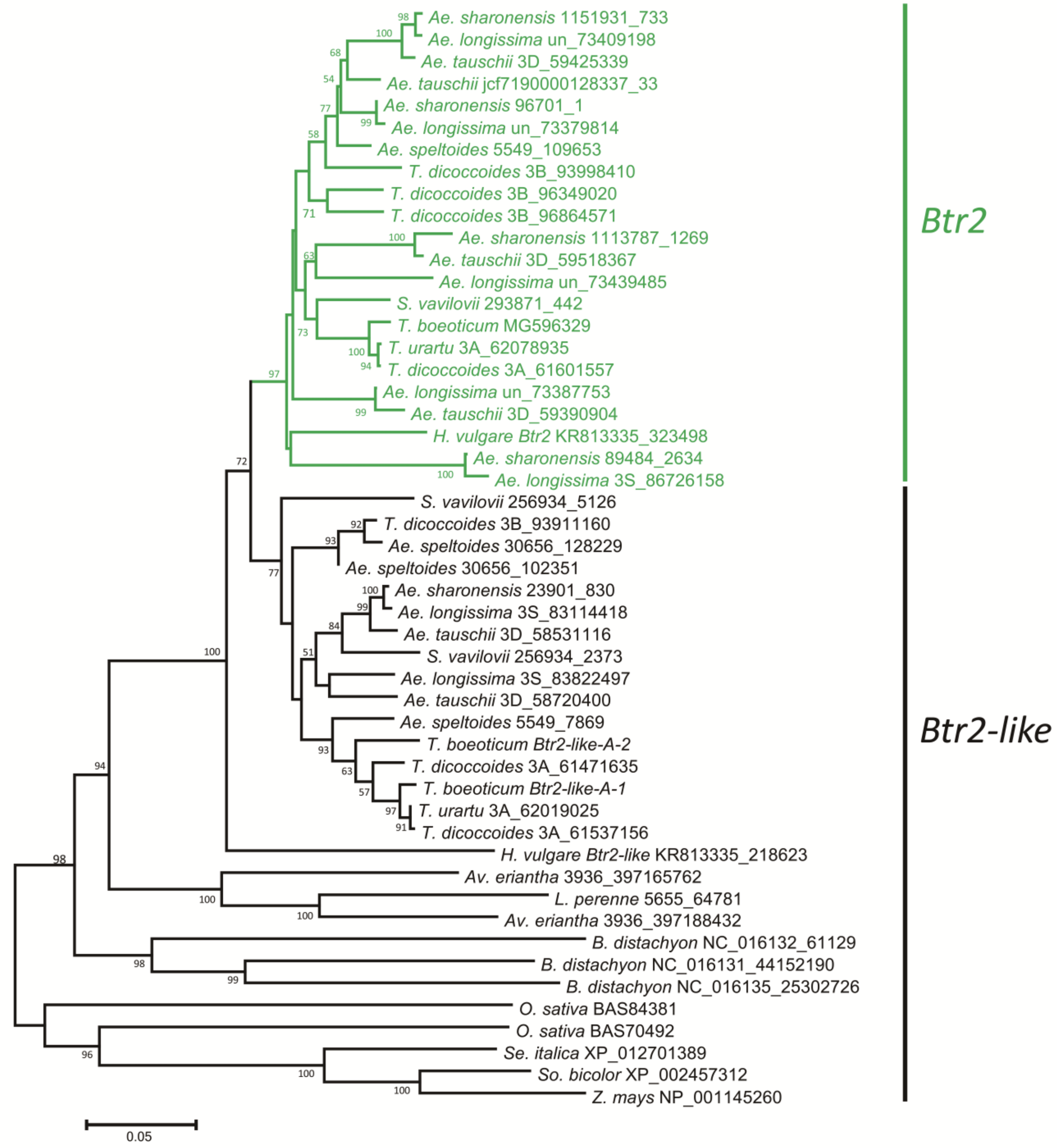

Fig. S7. Phylogenetic analysis of *Poaceae Btr2* and *Btr2-like* genes including those likely non-translated. Members of the *Btr2* clade are colored green. Numbers given at a branch node indicate bootstrap probabilities (only those >50% are shown). The number refer to the accession / scaffold / contig and the start location of each gene is given in Table S6.

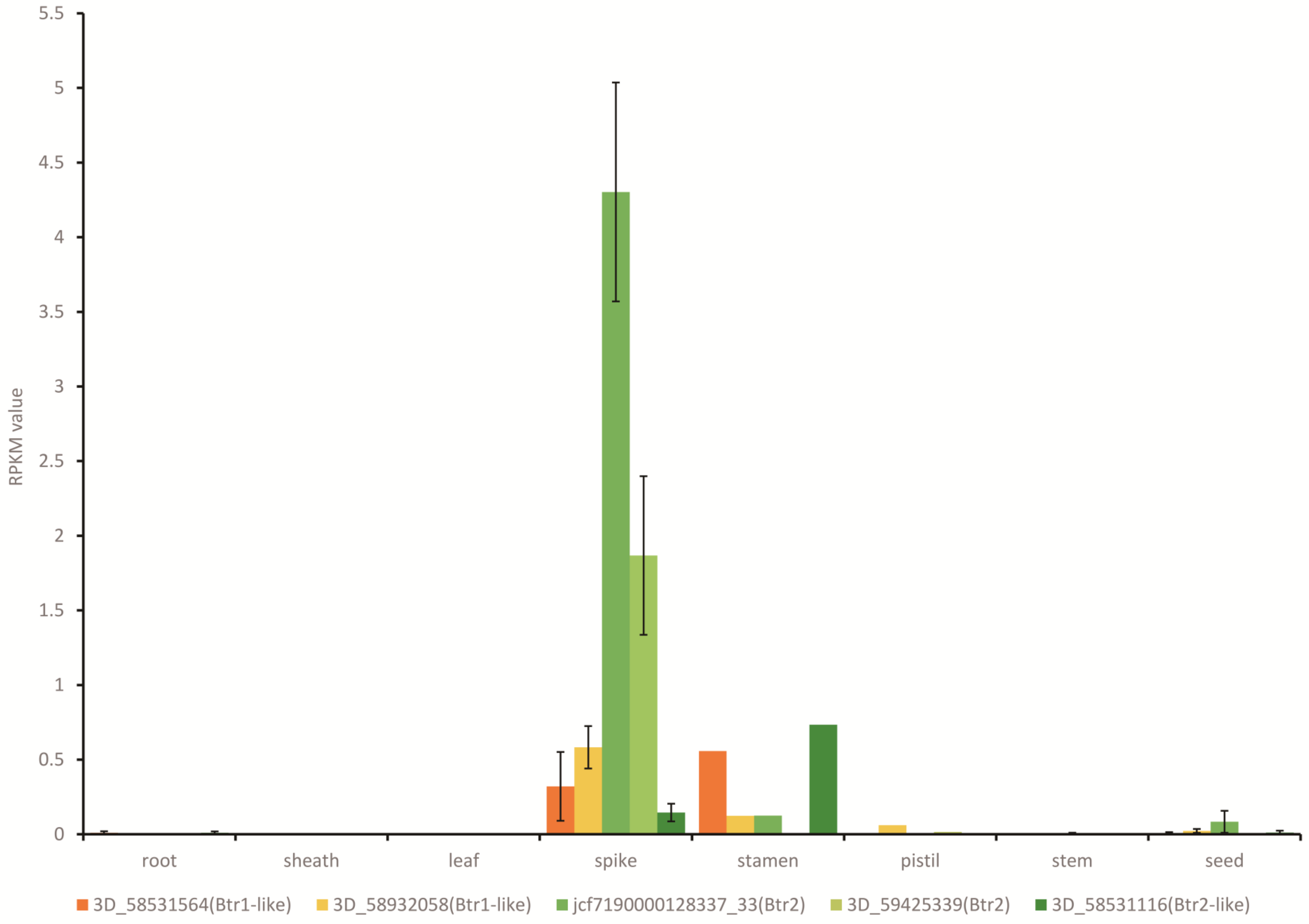

Fig. S8. RNA-seq derived transcript abundances (expressed as RPKM) of the *Ae. tauschii Btr* genes across various parts of the plant. The error bar indicates the SE (*n*=2 or 3).

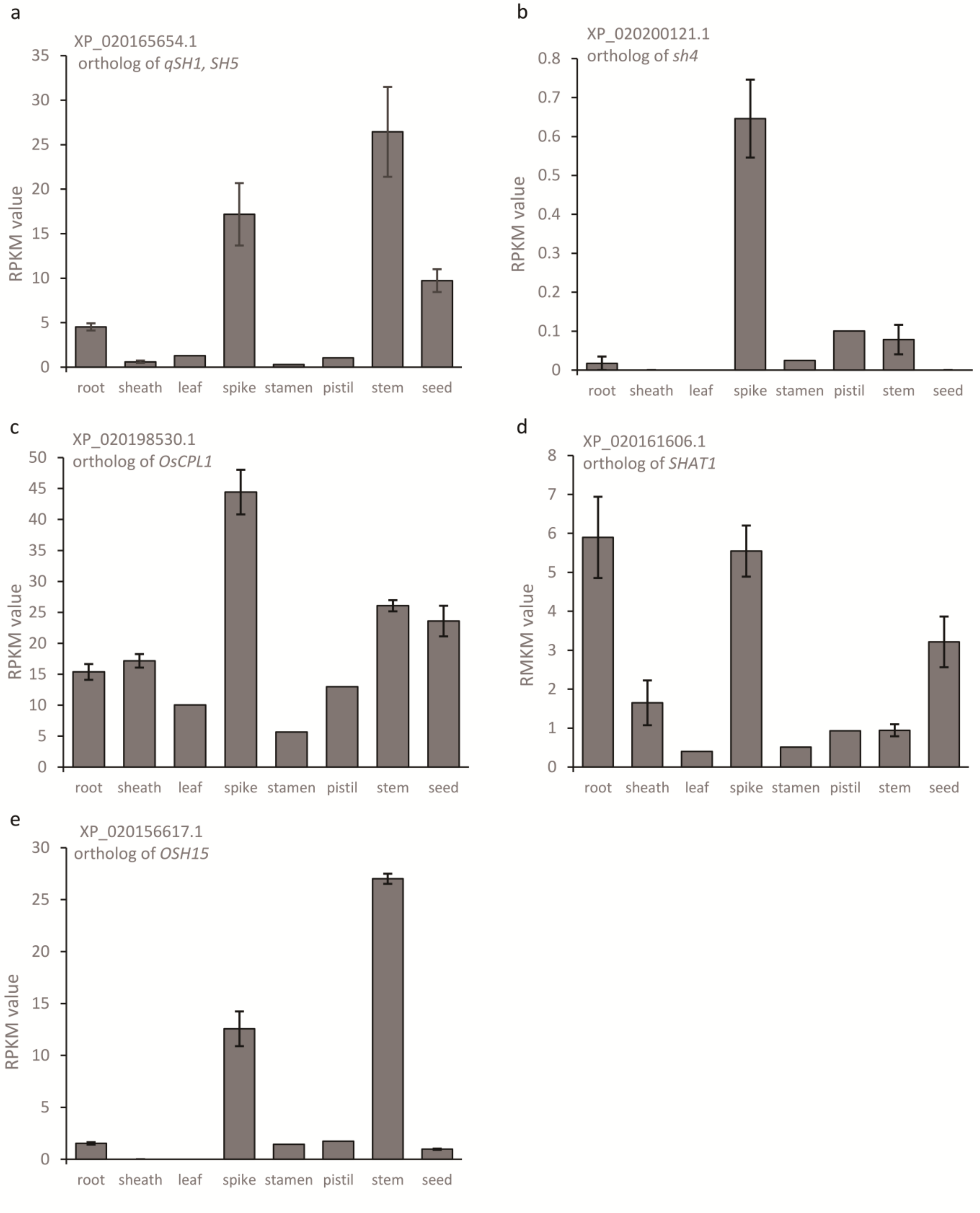

Fig. S9. RNA-seq derived transcript abundances (expressed as RPKM) of the *Ae. tauschii* orthologs of genes implicated in shattering in rice across various parts of the plant. (a) XP_020165654.1, the ortholog of *qSH1* and *SH5,* (b) XP_020200121.1, the ortholog of *sh4,* (c) XP_020198530.1, the ortholog of *OsCPL1,* (d) XP_020161606.1, the ortholog of *SHAT1,* (e) XP_020156617.1, the ortholog of *OSH15*. The error bar indicates the SE (n=2 or 3).
